# Emergence of an Auxin Sensing Domain in Plant-Associated Bacteria

**DOI:** 10.1128/mbio.03363-22

**Published:** 2023-01-05

**Authors:** José A. Gavira, Miriam Rico-Jiménez, Álvaro Ortega, Natalia V. Petukhova, Dmitrii S. Bug, Albert Castellví, Yuri B. Porozov, Igor B. Zhulin, Tino Krell, Miguel A. Matilla

**Affiliations:** a Laboratory of Crystallographic Studies, IACT (CSIC-UGR), Armilla, Spain; b Department of Biotechnology and Environmental Protection, Estación Experimental del Zaidín, Consejo Superior de Investigaciones Científicas, Granada, Spain; c Department of Biochemistry and Molecular Biology B and Immunology, Faculty of Chemistry, University of Murcia, Regional Campus of International Excellence Campus Mare Nostrum, Murcia, Spain; d Bioinformatics Research Center, Pavlov First Saint Petersburg Medical State University, St. Petersburg, Russia; e Molecular Biology Institute of Barcelona, CSIC, Barcelona, Spain; f The Center of Bio- and Chemoinformatics, I. M. Sechenov First Moscow State Medical University, Moscow, Russia; g Department of Microbiology, The Ohio State University, Columbus, Ohio, USA; University of Wisconsin—Madison

**Keywords:** LysR, signal transduction, antagonist, antibiotic, auxin, indole-3-acetic acid, ligands, protein evolution, sensor domain, signal sensing, structural biology, transcriptional regulator

## Abstract

Bacteria have evolved a sophisticated array of signal transduction systems that allow them to adapt their physiology and metabolism to changing environmental conditions. Typically, these systems recognize signals through dedicated ligand binding domains (LBDs) to ultimately trigger a diversity of physiological responses. Nonetheless, an increasing number of reports reveal that signal transduction receptors also bind antagonists to inhibit responses mediated by agonists. The mechanisms by which antagonists block the downstream signaling cascade remain largely unknown. To advance our knowledge in this field, we used the LysR-type transcriptional regulator AdmX as a model. AdmX activates the expression of an antibiotic biosynthetic cluster in the rhizobacterium Serratia plymuthica. AdmX specifically recognizes the auxin phytohormone indole-3-acetic acid (IAA) and its biosynthetic intermediate indole-3-pyruvic acid (IPA) as signals. However, only IAA, but not IPA, was shown to regulate antibiotic production in *S. plymuthica*. Here, we report the high-resolution structures of the LBD of AdmX in complex with IAA and IPA. We found that IAA and IPA compete for binding to AdmX. Although IAA and IPA binding does not alter the oligomeric state of AdmX, IPA binding causes a higher degree of compactness in the protein structure. Molecular dynamics simulations revealed significant differences in the binding modes of IAA and IPA by AdmX, and the inspection of the three-dimensional structures evidenced differential agonist- and antagonist-mediated structural changes. Key residues for auxin binding were identified and an auxin recognition motif defined. Phylogenetic clustering supports the recent evolutionary emergence of this motif specifically in plant-associated enterobacteria.

## INTRODUCTION

Bacteria have developed multiple strategies to sense and respond to environmental changes in order to efficiently adapt to specific ecological niches. These microorganisms possess a large number of proteins involved in signal transduction, and some bacteria can devote more than 12% of their genomes to these systems (1). The number of signal transduction proteins in environmental bacteria is particularly high (1–3), suggesting that the ability to adapt and respond to a broader diversity of signals is of particular relevance in environmental microorganisms. Input into signal transduction pathways is provided by sensor proteins, including transcriptional regulators, chemoreceptors, and sensor histidine kinases, that bind internal and external signals, typically through specialized ligand binding domains (LBDs) (4, 5). The diversity of signal molecules recognized by sensor proteins is broad (4), and ligand binding typically serves as the molecular stimulus that mediates the generation of the signaling output.

Transcriptional regulators (TRs) are the most abundant family of signal transduction systems in bacteria, and their abundance frequently exceeds 5% of the total number of proteins encoded in a genome (2, 5). In general, TRs consist of a DNA binding domain (DBD) and an LBD (2, 6), although there are TRs that do not contain a dedicated LBD or, alternatively, possess domains involved in protein-protein interactions (2, 4–6). Analysis of 761 bacterial and archaeal genomes resulted in the identification of a wide diversity of TRs that belong to 19 different families (6). Among these families, LysR-type transcriptional regulators (LTTRs) account for ~14% of the total number of TRs and constitute one of the largest families of these regulatory proteins (6). LTTRs modulate many different biological processes, including metabolism, transport, motility, cell division, antibiotic synthesis, exopolysaccharide production, and stress responses, among others (4, 7–9), and some bacterial genomes encode over 100 LTTR family members (2, 10), suggesting their pivotal role in regulating bacterial physiology and metabolism.

LTTRs consist of an N-terminal helix-turn-helix DBD connected through a flexible linker to a C-terminal LBD. Although LTTR LBDs are poorly conserved at their amino acid sequence level, they are relatively well conserved in structure (7–11)—as exemplified by various three-dimensional (3D) structures available for full-length LTTRs (9, 12–15) or their individual LBDs (6, 10, 11, 16–18). LTTR-LBDs consist of two α/β subdomains, RD-I and RD-II, that are linked by two antiparallel β-strands to form a cleft between the two subdomains that corresponds to the ligand binding site (7–10). Ligand binding generally induces a conformational change in the LBD that is transmitted to the DBD to ultimately alter its capacity to bind to DNA (7, 9, 10, 17, 18). Specific ligands have been identified for only a small fraction of LTTRs. By December 2022, over 5.8 million LTTRs were available in the NCBI protein database, but signal molecules have been experimentally identified for only ~70 LTTRs (4). These ligands include amino acids, organic acids, aromatic compounds, fatty acids, antibiotics, second messengers, and inorganic nutrients, among others (4). Knowledge on the signal molecules that are recognized by LTTRs is essential to understand the regulatory circuits and processes that are controlled by this abundant family.

Although it is generally believed that signal binding at LBDs triggers a physiological response, there has been an increasing number of reports on ligands that bind to LBDs of members of the main classes of signal transduction receptors without eliciting a response. For example, many ligands were identified that bind to the LBDs of the sensor kinases TodS and TmoS that did not modulate autophosphorylation and, as a consequence, gene expression (19, 20). Similarly, chemoreceptors Tar, CtpM, and TlpA bind some ligands that do not induce a chemotactic response (21–23). Furthermore, the transcriptional activity of some TRs, including CviR, NodD1, and PqsR, was antagonized by the binding of specific ligands (24–26). Signal agonists and antagonists compete with each other for protein binding, and consequently, these regulatory systems are controlled by the concerted action of these molecules. It is unclear as to whether sensing of signal antagonists has to be considered an artifact of evolution or whether it reflects a physiological role in bacteria.

In a previous study, we reported that the LTTR AdmX from the rhizosphere isolate Serratia plymuthica A153, a biocontrol bacterium that produces a broad spectrum of antibiotics (27), may be controlled by agonists and antagonists. AdmX is a pathway-specific LTTR that acts as a transcriptional activator of the expression of the gene cluster responsible for the biosynthesis of the hybrid nonribosomal peptide/polyketide antibiotic andrimid (28, 29). AdmX recognizes indole-3-acetic acid (IAA), a key auxin phytohormone essential for plant growth and development (30–32), with an affinity that is about 60 times lower than that of its biosynthetic intermediate indole-3-pyruvic acid (IPA). However, it was only IAA, and not IPA, that modulated andrimid production in *S. plymuthica* A153 (29). In addition, IAA and IPA binding to AdmX resulted in very different thermodynamic profiles, and furthermore, both compounds caused dissimilar changes to the secondary structure of AdmX (29).

The structural reasons that determine whether a compound behaves as an agonist and antagonist are largely unknown. To advance this knowledge, we report here the high-resolution structures of AdmX-LBD in the presence of IAA and IPA, and identify different conformational changes resulting from IAA and IPA binding. Key residues for auxin binding were determined, and multidisciplinary approaches allowed for a deeper understanding of the evolutionary processes that resulted in the emergence of auxin sensing domains in plant-associated bacteria.

## RESULTS

### IAA and IPA compete for binding to AdmX-LBD.

**FIG 1:**
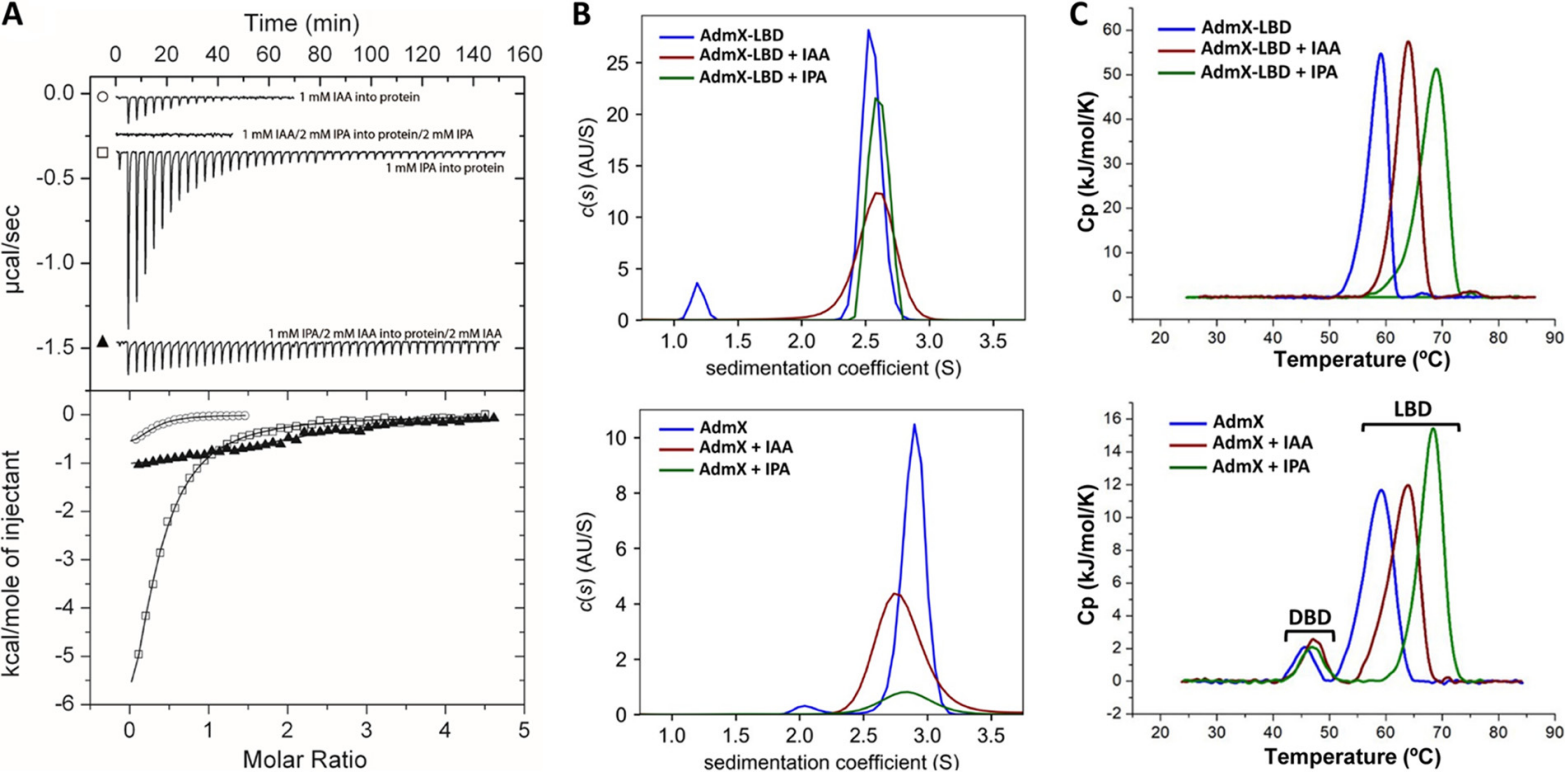
Biophysical characterization of the interaction of indole-3-acetic acid (IAA) and indole-3-pyruvic acid (IPA) with AdmX and AdmX-LBD. (A) IAA and IPA compete for binding at AdmX-LBD. Shown are the results from isothermal titration calorimetry analysis of the binding of IAA and IPA to AdmX-LBD in the presence and absence of saturating concentrations of IPA and IAA, respectively. (Upper panel) Titration raw data for the injection of 6.4- to 9.6-μL aliquots of 1 mM IAA or IPA into 50 to 100 μM AdmX-LBD in the absence and presence of 2 mM IPA or IAA (present in both the injector syringe and sample cell). (Lower panel) Integrated, dilution heat-corrected and concentration-normalized peak areas fitted with the “One binding site” model of ORIGIN. (B) Sedimentation velocity analytical ultracentrifugation analysis of AdmX and AdmX-LBD in the absence and presence of IAA or IPA. Values correspond to experiments conducted at 10°C in the corresponding buffers for AdmX (50 mM KH_2_PO_4_-K_2_HPO_4_, 300 mM NaCl, 10% [vol/vol] glycerol, 2 mM β-mercaptoethanol [pH 7.0]) and AdmX-LBD (20 mM HEPES, 150 mM NaCl, 2 mM β-mercaptoethanol [pH 7.4]). (C) Effect of IAA and IPA binding on the thermal unfolding behavior of AdmX-LBD and AdmX. Differential scanning calorimetry thermograms are shown. LBD, ligand binding domain; DBD, DNA binding domain.

Given the structural similarity between IAA and IPA, as well as their different effects on andrimid biosynthesis (29), we explored their role as agonists and antagonists, respectively, by analyzing their capacity to compete for binding at AdmX-LBD through microcalorimetric titrations. First, we analyzed IAA binding to AdmX-LBD in the absence and presence of saturating concentrations of IPA. Our data revealed that in the presence of IPA, no IAA binding to AdmX-LBD occurred (Fig. 1A). We subsequently evaluated IPA binding to AdmX-LBD in the presence of saturating concentrations of IAA. Although we found that AdmX-LBD still bound IPA, heat released from this binding was drastically reduced in the presence of IAA (Fig. 1A). Taken together, these data show that both auxins compete for binding at AdmX-LBD.

### IAA and IPA binding does not alter the oligomeric state of AdmX.

LTTRs are typically homotetrameric proteins (7, 9), but this oligomeric state is not universal and some members of the family are found in lower (e.g., dimer) and higher (e.g., octamer) oligomeric states (9, 14, 33, 34). Ligand binding was also shown to modulate the oligomeric state of some LTTRs (7, 33). To investigate the oligomeric states of AdmX and AdmX-LBD and the influence of auxin binding on oligomerization, both proteins were analyzed by sedimentation velocity analytical ultracentrifugation (AUC) in the absence and presence of saturating concentrations of IAA and IPA. To understand the different species derived from the AUC assays, the protein structures of AdmX and AdmX-LBD were modeled with AlphaFold 2 (35) and the theoretical sedimentation coefficients were calculated using the modeling software HYDROPRO (36). The theoretical sedimentation values (*s*_20,_*_w_*) calculated for AdmX-LBD were 2.6 S and 4.3 S for the monomer and dimer, respectively, whereas theoretical *s*_20,_*_w_* values were 2.7 S, 4.3 S, and 6.8 S for the AdmX monomer, dimer, and tetramer, respectively. AUC analyses of ligand-free AdmX-LBD showed a predominant species, 3.6 S, which is between the theoretical *s*_20,_*_w_* values for the monomeric and dimeric species, reflecting a fast equilibrium between the monomeric and the dimeric species, that is shifted toward the dimeric state (Fig. 1B; note that Fig. 1B shows the sedimentation coefficient values recorded in buffer, whereas theoretical sedimentation coefficients, [*s*_20,_*_w_*], are standard values normalized for migration in water at 20°C.) No significant shifts in the sedimentation behavior were detected in the presence of IAA or IPA (Fig. 1B). AUC runs were subsequently conducted with AdmX. No differences were observed in the oligomeric state of AdmX at different concentrations of the protein: between 5 and 20 μM (not shown). At the concentration assayed (27 μM), a predominant oligomeric species for AdmX was observed, with a sedimentation coefficient (*s*_20,_*_w_*) of 6.3 S (Fig. 1B), which is close to the tetrameric state. No significant alteration in the autoassociation equilibrium was observed in the presence of IAA and IPA, with *s*_20,_*_w_* values of 6.3 S and 6.1 S, respectively (Fig. 1B).

### Interdomain communication in AdmX in the presence of IAA and IPA.

Ligand binding to the LTTR-LBD typically creates a molecular stimulus that is transmitted to the DBD (7, 9, 10). We hypothesized that the differences in activity of AdmX in the presence of IAA and IPA may be due to a difference in interdomain communication. In a previous study of the two-domain transcriptional regulator TtgV, we found that differential scanning calorimetry (DSC) is a convenient technique to study interdomain communication in TRs (37). Therefore, we conducted DSC assays to investigate the thermal unfolding of AdmX and AdmX-LBD in the presence and absence of saturating concentrations of IAA and IPA.

The thermogram of AdmX-LBD consists of a single endothermic unfolding event that shifts from a midpoint temperature (*T_m_*) of unfolding of 59.1°C in the absence of ligands to 63.9°C and 69.0°C in the presence of IAA and IPA, respectively (Fig. 1C and see Table S1 in the supplemental material). We subsequently analyzed the unfolding characteristics of the full-length protein. DSC analyses of AdmX showed two unfolding events centered at 45.5°C and 59.3°C, representing unfolding of the DBD and LBD, respectively (Fig. 1C and Table S1). Whereas the addition of IAA and IPA caused a stabilization of the LBD that was comparable to that of the individual domain, the addition of ligands had a very similar effect on the DBD, with increases in *T_m_*s of 1.7°C and 1.2°C upon the addition of IAA and IPA, respectively (Fig. 1C and Table S1). The data thus indicate that binding of ligands to the LBD is transmitted to the N-terminal DBD and that the differences in the activity of AdmX in the presence of IAA and IPA are not reflected in major changes in interdomain communication.

10.1128/mbio.03363-22.6 TABLE S1 Thermodynamic parameters derived from differential scanning calorimetry and microcalorimetric titrations. Download Table S1, DOCX file, 0.02 MB. Copyright © 2023 Gavira et al. 2023 Gavira et al. https://creativecommons.org/licenses/by/4.0/ This content is distributed under the terms of the Creative Commons Attribution 4.0 International license.

The magnitude of enthalpy changes (peak area) agreed with the magnitude of increase in *T_m_* (peak summit). LBD enthalpy changes of AdmX and AdmX-LBD in complex with IPA were superior to those of the IAA complexes (Table S1). In contrast, the enthalpy change of the DBD was superior in the AdmX/IAA complex compared to the AdmX/IPA complex (Table S1), possibly due to subtle changes in interdomain communication.

### The binding sites of IAA and IPA are atypical for LTTR.

**FIG 2:**
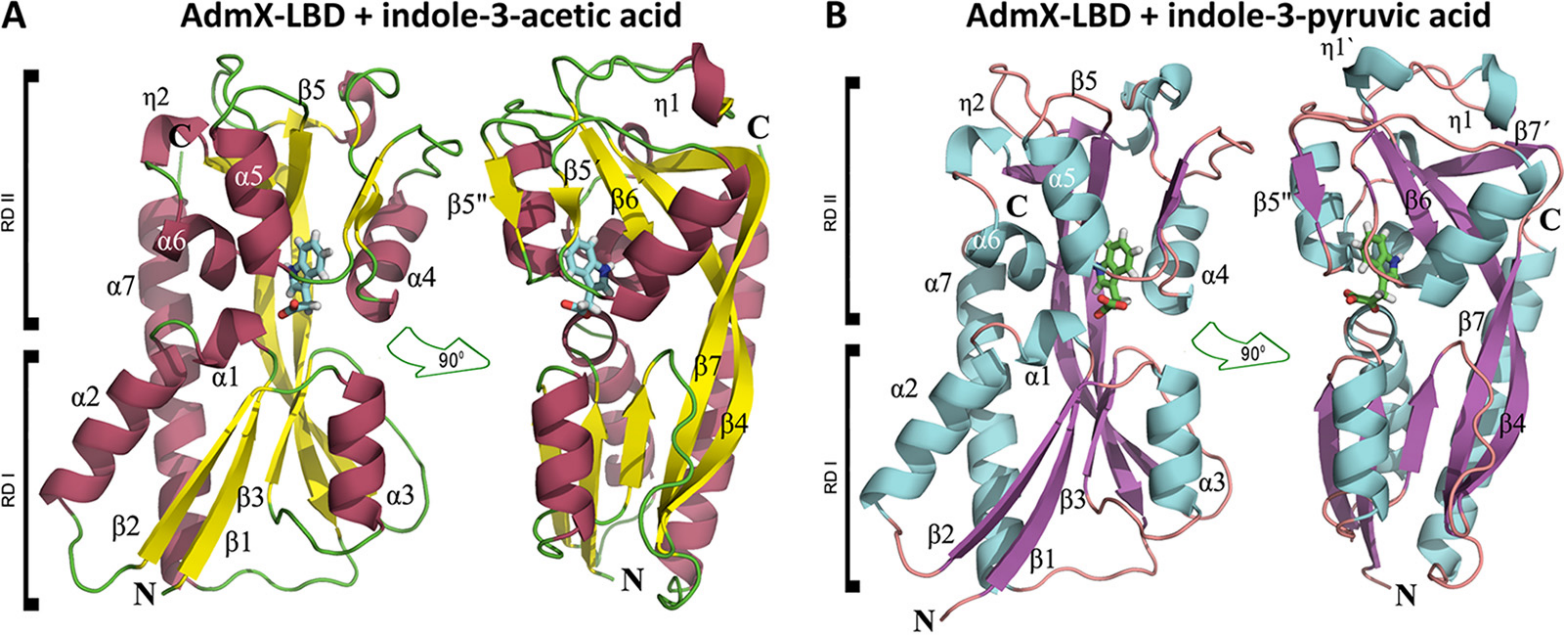
Three-dimensional structures of the AdmX-LBD in complex with IAA and IPA. Shown is a ribbon diagram of AdmX-LBD bound to IAA (A) and IPA (B) in which secondary structural elements are labeled. Each structure is shown in two different orientations, rotated by 90°. IAA and IPA are shown in the stick mode in cyan and green, respectively.

To investigate the molecular bases of auxin recognition by AdmX, we attempted to determine the three-dimensional structure of full-length AdmX. However, the low purification yield and the protein instability prevented its crystallization. We subsequently conducted crystallization trials of AdmX-LBD in its ligand-free form and in complex with IAA and IPA. AdmX-LBD crystals were obtained in the presence of IAA and IPA, and the structures were determined to resolutions of 1.81 Å and 2.25 Å in the P2_1_2_1_2 and P2_1_2_1_2_1_ space groups, respectively (Fig. 2 and Table S2). In accordance with the dimeric state of AdmX-LBD in solution (Fig. 1B), and similarly to other members of the LTTR family (7, 9, 10), the asymmetric unit contains an AdmX-LBD dimer in both polymorphs, but in the case of AdmX-LBD the second monomer position corresponds to a 180° rotation along the axis of the α7 helix followed by an almost 90° rotation perpendicular to the helix main axis (Fig. S1A and B).

10.1128/mbio.03363-22.1 FIG S1 Dimer configurations of the asymmetric unit of AdmX-LBD bound to indole-3-acetic acid (A) and indole-3-pyruvic acid (B). (A and B) Chains A are shown in green (IAA complex) and salmon (IPA complex), whereas chains B are shown in dark blue (IAA complex) and light blue (IPA complex). β-Strands are represented in yellow to show the alignment of both β-strands within the dimer. IAA and IPA are depicted in ball-mode in carbon-cyan and carbon-green, respectively. (C) Superposition of the Cα atoms of the structures of AdmX-LBD bound to IAA (red) and IPA (green). Shown is a zoom at the ligand binding pocket. (D and E) Mesh-ribbon and space-filling representation of the AdmX-LBD dimers’ interface in complex with IAA (D) and IPA (E). Chains A and B and residues at the interface for each chain are colored in cyan and blue and green and red, respectively. Download FIG S1, JPG file, 1.3 MB. Copyright © 2023 Gavira et al. 2023 Gavira et al. https://creativecommons.org/licenses/by/4.0/ This content is distributed under the terms of the Creative Commons Attribution 4.0 International license.

10.1128/mbio.03363-22.7 TABLE S2 Data collection and refinement statistics of the different structures of AdmX-LBD. Statistics for the highest-resolution shell are shown in parentheses. Download Table S2, DOCX file, 0.01 MB. Copyright © 2023 Gavira et al. 2023 Gavira et al. https://creativecommons.org/licenses/by/4.0/ This content is distributed under the terms of the Creative Commons Attribution 4.0 International license.

AdmX-LBD exhibits the classical fold observed for the LBDs of LTTRs (7, 9–11, 15, 38), which consists of two α/β subdomains, RD-I and RD-II, composed of αβα sandwiches with a Rossmann-like topology (Fig. 2). The RD-I subdomain is formed by three β-strands (β1, β2, and β3) and three α-helices (α1, α2, and α3), whereas the RD-II subdomain has three β-strands (β6, β7, β8), three α-helices (α4, α5, α6), and the two 3_10_-helices (η1 and η2) (Fig. 2). Both subdomains are connected by a hinge region formed by β4, β9, and α7, crossing and participating in both domains (Fig. 2 and Fig. S1A and B). While in each subdomain the β-strands run in a parallel fashion, β4 and β9 run antiparallel. This architecture is reminiscent of the fold observed in extracellular solute binding proteins (39, 40), and despite the low sequence identity between AdmX-LBD and other LTTRs, its structure resembles those of the LBDs of other crystalized members of the LTTR family (7, 9–11, 15, 38). Indeed, structural alignments of AdmX-LBD using the DALI algorithm (41) revealed that the closest homologs are sensor domains of LTTRs and that AdmX-LBD is most similar to the LBD of the octopine-responsive LTTR OccR from the plant-pathogenic bacterium Agrobacterium tumefaciens (PDB ID 5VVH; Z-score, 21.9; RMSD, 3.0 Å) (Table 1). However, the amino acid identity between AdmX and the 20 closest structures is only between 8% and 20% (Table 1).

**TABLE 1:** Structural alignment of the AdmX-LBD in complex with IAA with structures deposited in the Protein Data Bank^*a*^

PDB ID	Name	Ligand(s)	Bacterial species	Z-score^*b*^	RMSD^*b*^	lali^*b*^	nres^*b*^	% identity
5VVH	OccR	Octopine	Agrobacterium tumefaciens	21.9	3.0	197	210	15
5TED	QuiR	Shikimate	Listeria monocytogenes	20.1	2.4	191	210	20
3HFU	CynR	Cyanate, azide	Escherichia coli	19.6	3.0	191	204	14
5TPI	YneJ		Klebsiella pneumoniae	19.5	2.8	194	205	11
3HO7	OxyR	Hydrogen peroxide?	Porphyromonas gingivalis	19.0	2.5	194	220	13
2QL3	RHA1_ro01847		Rhodococcus jostii	18.3	2.5	184	205	16
3K1M	BenM	Benzoate, *cis,cis*-muconate	Acinetobacter baylyi	18.2	2.9	193	305	15
5Y2V	CcmR	2-Phosphoglycolate, 2-oxoglutarate, ribulose 1,5-bisphosphate	*Synechocystis* sp.	18.2	2.8	195	304	18
5MMH	AmpR	UDP-*N*-acetylmuramic acid-pentapeptides, *N*-acetyl-glucosamine-(1–4)-1,6-anhydro-*N*-acetylmuramic acid-pentapeptides, 1,6-anhydro-*N*-acetylmuramic acid-pentapeptides	Pseudomonas aeruginosa	17.8	2.7	189	206	13
6G1B	OxyR	Hydrogen peroxide	Corynebacterium glutamicum	17.8	3.0	198	326	13
3FXQ	TsaR	*para*-Toluensulfonate, sulfate	Comamonas testosteroni	17.7	2.7	190	296	14
6GZ2	LeuO		Escherichia coli	17.0	3.3	186	201	10
4LQ5	CysB	*N*-acetylserine, *O*-acetylserine, sulfide, thiosulfate, sulfate	Salmonella enterica serovar Typhimurium	16.6	2.6	190	241	8
6L33	MexT		Pseudomonas aeruginosa	16.5	2.8	179	183	10
3OXN	VP0027		Vibrio parahaemolyticus	16.4	3.0	193	215	11
4AB5	MetR		Neisseria meningitidis	16.3	3.6	192	220	12
4QBA	CcpE	Citrate	Staphylococcus aureus	16.1	3.2	176	204	17
5YDO	HypT	Hypochlorous acid	Salmonella enterica serovar Typhimurium	15.2	3.1	178	209	15

a Shown are the top 20 structures according to the Z-score. Alignments were made with the DALI server (41).

b lali, number of aligned positions; rmsd: root-mean-square-deviation of structurally equivalent C-alpha atoms in 3-D superimposition; nres: number of residues in the target structure; Z-score represents similarities between structures - higher Z-scores are more similar to the query.

**FIG 3:**
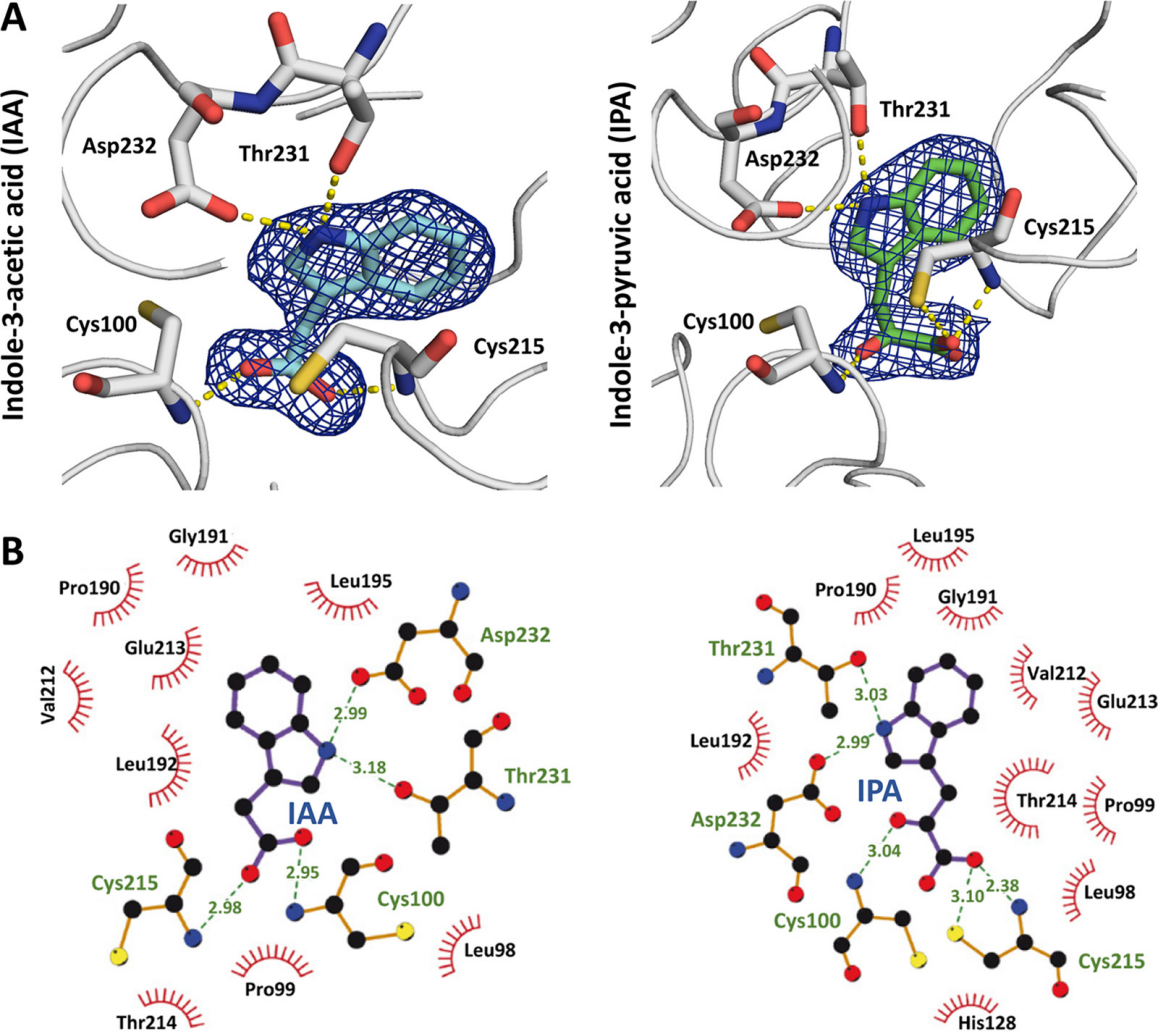
Molecular detail of auxin recognition by AdmX-LBD. (A) Enlarged views of the ligand binding site of AdmX. The meshes represent the final |2*F_o_* − *F_c_*| electron density map contoured at the 1.0-σ level for the complexes of AdmX-LBD with IAA and IPA. (B) Amino acid residues involved in auxin binding. Dashed lines indicate hydrogen bonds with distances provided in angstroms, whereas hydrophobic interactions are shown as spoked arcs. The figures were produced using PYMOL and LIGPLOT+. In all cases, cartoons correspond to chains A of both structures.

Well-defined electron density was identified for IAA and IPA, allowing thus the precise positioning of both ligands into the AdmX-LBD binding pocket and permitting the identification of residues involved in auxin binding (Fig. 2 and Fig. 3). One molecule of IAA or IPA was bound to each monomer (Fig. 2 and Fig. S1). In contrast to other members of the LTTR family, where effectors are generally accommodated in the hinge region between the RD-I and RD-II subdomains (9, 16, 38, 42–44), both IAA and IPA, bound to the RD-II subdomain (Fig. 2). IAA and IPA showed the same orientation in the ligand pocket (Fig. S1), and no major differences in the mode of auxin binding were observed between both monomers (Fig. 2 and 3).

### The molecular details of IAA and IPA binding to AdmX are very similar.

The indole moiety of IAA and IPA establishes hydrogen bonds with Thr231 and Asp232 through the nitrogen present in the pyrrole ring, but it is also coordinated by diverse hydrophobic interactions with a number of nonpolar and negatively charged amino acids (Pro190, Gly191, Leu192, Leu195, Val212, and Glu213) (Fig. 3). The acetic and pyruvic acid groups of IAA and IPA, respectively, form hydrogen bonds with Cys100 and Cys215 involving the carboxylate and ketone groups (Fig. 3).

**FIG 4:**
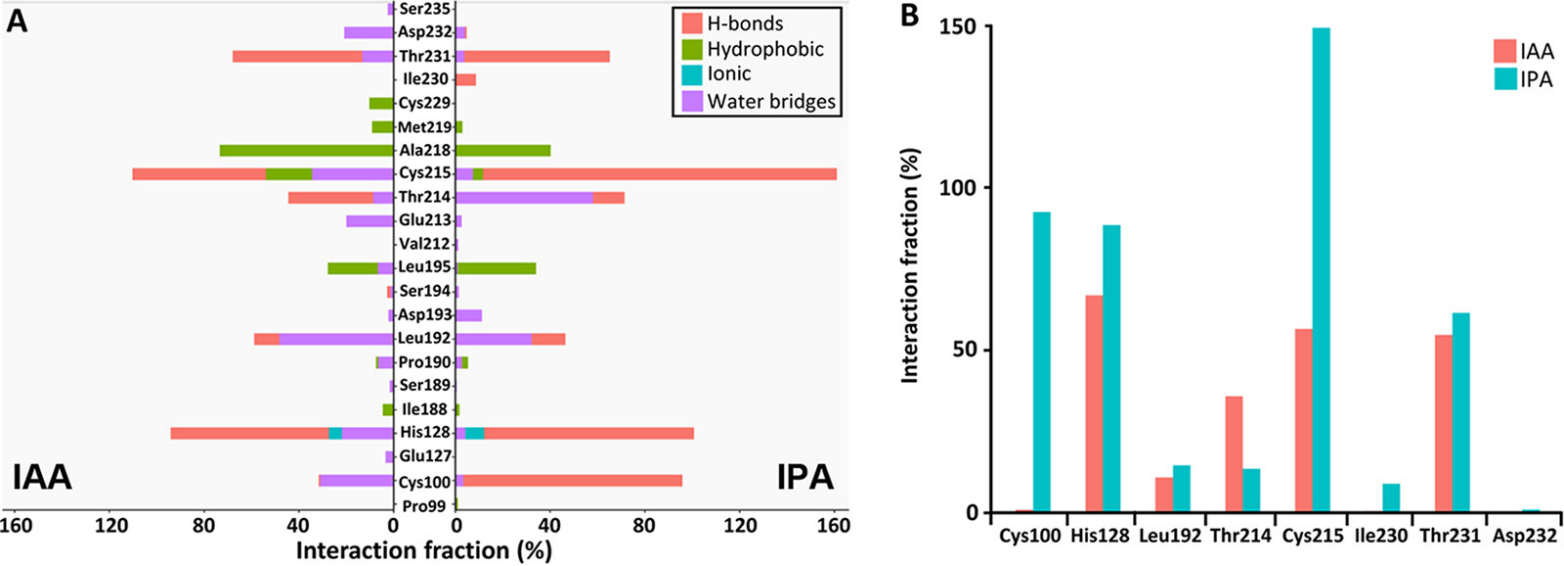
Amino acid residues involved in auxin binding determined by molecular dynamics simulations. (A) Frequency of occurrence of hydrogen bonds, hydrophobic and ionic interactions, and water bridges throughout 500-ns molecular dynamics simulations of AdmX-LBD with IAA and IPA bound. (B) Hydrogen bonding dynamics of AdmX with IAA and IPA bound throughout 500-ns molecular dynamics simulations. Residues with interaction fraction less than 0.5% were omitted from the plot.

Molecular dynamics (MD) simulations of the two auxin-bound structures were conducted to further investigate IAA and IPA binding, which allowed us to evaluate the hydrogen bond network and the hydrophobic and ionic interactions as well as water bridges’ formation (Fig. 4A and Fig. S2A). The frequency of hydrogen bond formation in MD simulations is shown in Fig. 4B. MD simulations revealed that 22 amino acids interact with IAA and IPA (Fig. 4A). Although both auxins establish similar interactions with key residues, a stronger hydrogen bond network was determined for IPA (Fig. 4), which is indicative of the higher binding affinity determined for this auxin (Fig. 1 and Table S1). Instead, a higher number of water bridges and hydrophobic interactions were observed for IAA (Fig. 4A). Consensus interactions with both ligands include Cys215 as the key residue, with significant H-bonding with the carboxyl groups of IAA and IPA during the MD simulations (Fig. 4 and Fig. S2). Cys100, His128, and Thr214 were additional coordinators for the carboxylate and ketone groups through the establishment of hydrogen bonds. Thr231 and Asp232 provide NH-pyrrole ring interactions, and Leu192 as well as Ala218 support hydrophobic coordination of the indole ring (Fig. 4 and Fig. S2). Taken together, MD simulations revealed thus significant differences in the binding modes of IAA and IPA to AdmX-LBD, including a higher frequency of hydrogen bridge formation between the side chains of IPA and Cys100, His128, and Cys215 (Fig. 4).

10.1128/mbio.03363-22.2 FIG S2 Analysis of AdmX-LBD by molecular dynamics simulations. (A) Amino acid residues involved in IAA and IPA binding determined by molecular dynamics simulations. Shown are the frequency of occurrence of hydrogen bonds, hydrophobic and ionic interactions, and water bridges throughout 500-ns molecular dynamics simulations of AdmX-LBD with IAA and IPA. (B) Comparison of protein chain stability during molecular dynamics 500-ns simulations of AdmX-LBD in complex with IAA and IPA ligands. Shown are a root mean square fluctuation (RMSF) plot showing local changes along chain B of AdmX-LBD and a plot showing the RMSF of Cα atoms for AdmX-LBD bound with IAA (blue) and IPA (orange) as derived from the MD simulation. Major local differences are highlighted by a red rectangle. Download FIG S2, JPG file, 0.7 MB. Copyright © 2023 Gavira et al. 2023 Gavira et al. https://creativecommons.org/licenses/by/4.0/ This content is distributed under the terms of the Creative Commons Attribution 4.0 International license.

### Evidence for differential agonist- and antagonist-induced structural changes.

**FIG 5:**
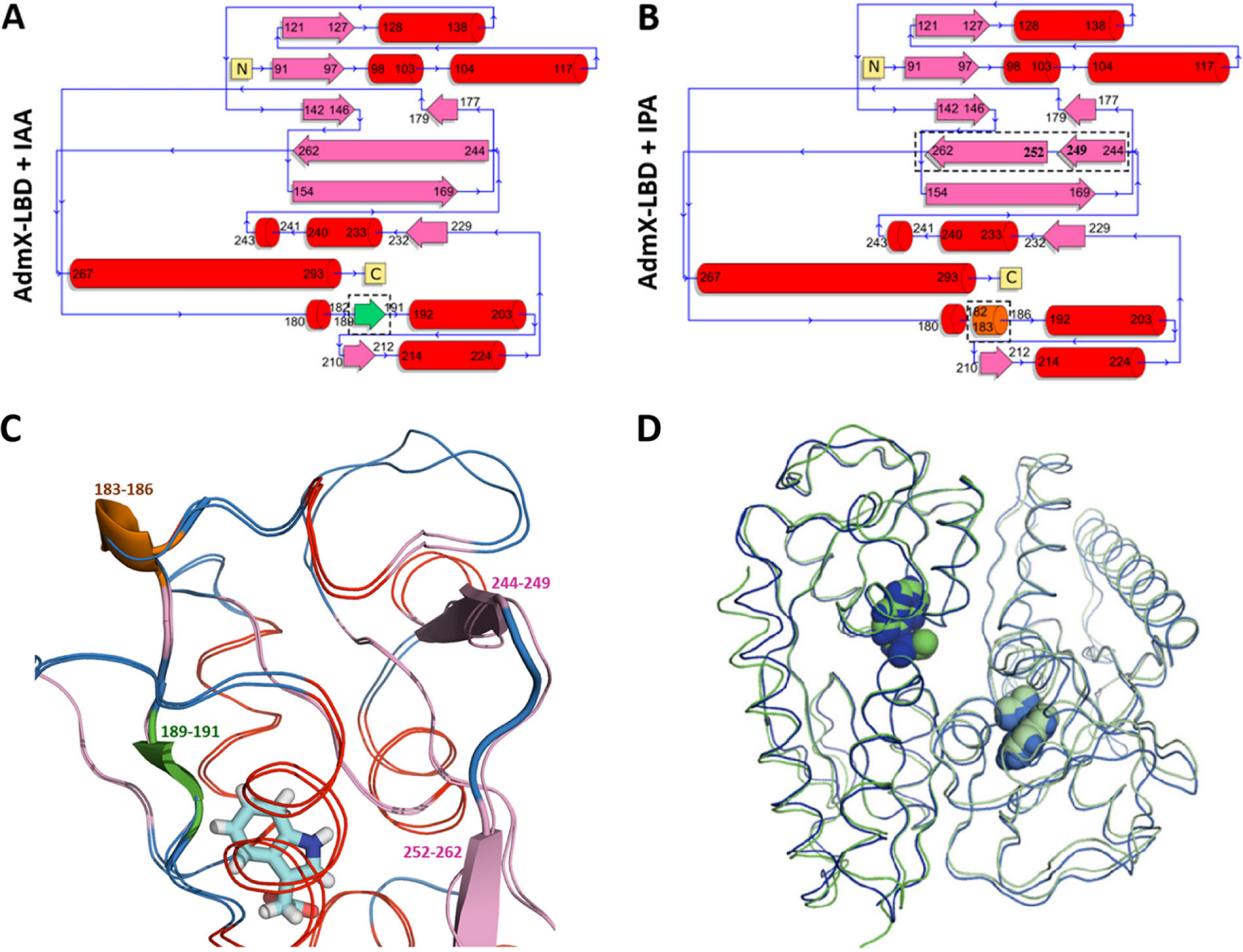
Structural changes in AdmX-LBD with IAA and IPA bound. (A and B) Topological organization of secondary structural elements of AdmX bounded to IAA (A) and IPA (B) as determined with DSSP (109). We observed how the small parallel β-sheets near the binding region of IAA (residues 189 to 191) are absent in the model with IPA, which on the other hand shows an α_10_-helix (residues 183 to 186) and the division of the long β-sheet number 7 (residues 244 to 262) in two fragments. (C) Superimposition of both structural models represented as ribbons and colored in red (α-helixes), pink (β-sheets), and blue (turns). New or modified secondary structure elements are shown in cartoon mode. For simplicity, only IAA is represented in stick mode. (D) Superposition of the AdmX-LBD homodimers with IAA (green) and IPA (blue) bound. Auxins are shown in space-filling mode.

AdmX-LBD crystals in complex with IAA and IPA showed a slightly different unit cell (Table S2), revealing a different packing. The crystallographic homodimers of the asymmetric units of the IAA and IPA structure superposed with a root-mean-square deviation (RMSD) for Cα atoms of 0.89 Å, indicating slight structural differences (Fig. 5). When both chains of the same structure were superimposed, RMSD values of 0.78 Å and 0.50 Å for the IAA and IPA, respectively, were obtained (Table S3), indicating that the structural differences between different structures are greater than the differences between the chains of the same structure.

10.1128/mbio.03363-22.8 TABLE S3 Root-mean-square displacement (RMSD) values (in angstroms) for Cα atoms of individual chains of AdmX-LBD in complex with IAA and IPA. Download Table S3, DOCX file, 0.01 MB. Copyright © 2023 Gavira et al. 2023 Gavira et al. https://creativecommons.org/licenses/by/4.0/ This content is distributed under the terms of the Creative Commons Attribution 4.0 International license.

Several structural changes were identified between both polymorphs. First, the analysis of the secondary elements of AdmX-LBD with IAA and IPA revealed the absence in the IPA structure of the small β-strand located in the vicinity of the auxin binding region (residues 189 to 191) (Fig. 2 and Fig. 5A to C). In contrast, a short α_10_-helix (residues 183 to 186) was formed in the structure of AdmX-LBD in complex with IAA (Fig. 2 and Fig. 5A to C). In accordance with this piece of data, root mean square fluctuation (RMSF) measures, indicative of the average deviation of a particular amino acid residue from a reference position over the course of the MD simulations, revealed that highest differences were observed at the region including residues 168 to 215, with greater fluctuations in the presence of IPA (Fig. S2B). Second, the long β-strand 7 (residues 244 to 262) in AdmX-LBD with IAA bound is broken into two shorter β-strands (residues 244 to 249 and 252 to 262) in the presence of IPA (Fig. 2 and Fig. 5A to C). Third, important differences were observed in the dimer interfaces. There was a lower number of salt bridges between AdmX-LBD monomers in the AdmX-LBD/IAA structure, 2, than in the IPA structure, 6 (Table S4). The PISA software (45) revealed a higher stabilization energy upon dimerization in the AdmX-LBD crystals in complex with IPA (−17.1 kcal/mol) than in the complex with IAA bound (−13.7 kcal/mol). The total number of interactions (e.g., hydrogen bonds and salt bridges) is much lower in the IAA than in the IPA structure, 14 versus 32, respectively (Table S4), indicating a more tightly packed dimer in the presence of IPA, which also suggests a reduction in the flexibility of the dimer. Accordingly, a 3.3% reduction in the total surface area of the AdmX-LBD dimer was observed with IPA bound (surface area, 17,200 Å^2^) with respect to that in the IAA complex (surface area, 17,760 Å^2^) (Table S4), showing a very similar interface region in both cases (Fig. S1D and E).

10.1128/mbio.03363-22.9 TABLE S4 Analysis of the AdmX-LBD dimer interface statistics obtained from PDBsum and PISA. Download Table S4, DOCX file, 0.01 MB. Copyright © 2023 Gavira et al. 2023 Gavira et al. https://creativecommons.org/licenses/by/4.0/ This content is distributed under the terms of the Creative Commons Attribution 4.0 International license.

### Defining an auxin binding motif in LTTRs.

**FIG 6:**
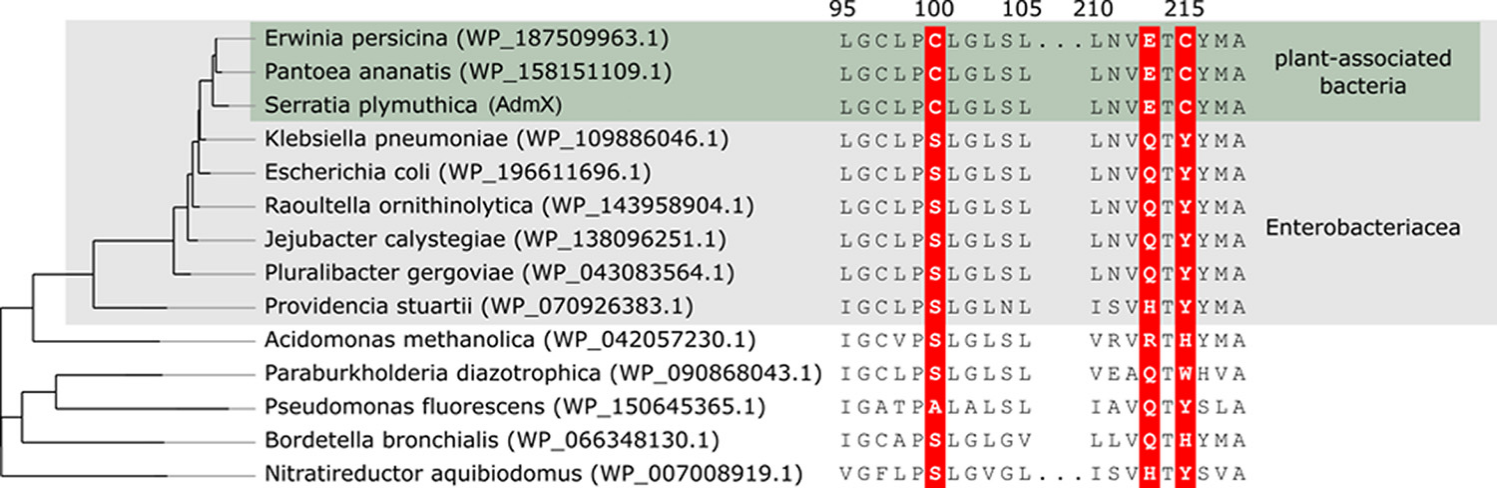
Protein sequence alignments and maximum likelihood tree from representative AdmX homologs. Residues Cys100, Glu213, and Cys215 are highlighted for purposes of comparison.

**FIG 7:**
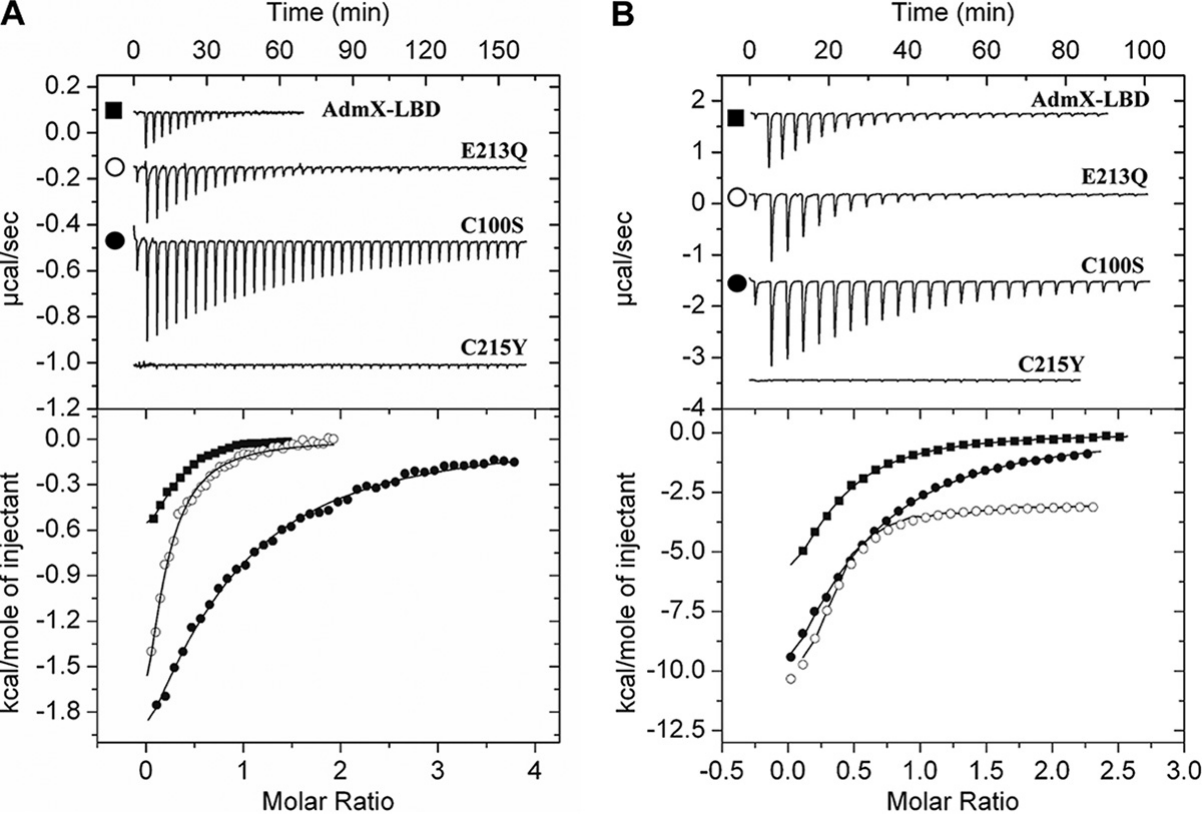
Isothermal titration calorimetry studies of AdmX-LBD and site-directed mutants. (A and B) Microcalorimetric titrations with indole-3-acetic acid (A) and indole-3-pyruvic acid (B). Protein at 50 to 100 μM was titrated with 6.4- to 12.8-μL aliquots of 1 to 5 mM IAA and 1 to 3 mM IPA. (Upper panels) raw titration data. (Lower panels) integrated, dilution heat-corrected and concentration-normalized peak areas of the titration data fitted with the “One binding site” model of ORIGIN. The derived dissociation constants are shown in Table S1.

AdmX-LBD fails to bind both indole and tryptophan (29), which strongly suggests that the recognition of the acetic and pyruvic acid side chains of IAA and IPA, respectively, is key for auxin sensing. Crystal structures (Fig. 2 and 3) and MD simulations (Fig. 4 and Fig. S2) revealed that Cys100 and Cys215 establish hydrogen bonds with the carboxylate and ketone groups of IAA and IPA side chains. We used AdmX-LBD as a query for a BLAST search against the entire RefSeq protein database. Over 1,560 protein sequences were collected based on coverage and identity, and subsequent multiple-sequence alignments revealed that the residues Cys100 and Cys215 were exclusively conserved in 13 AdmX homologs from bacteria of the *Serratia*, *Pantoea*, and *Erwinia* genera (Fig. 6 and Fig. S3 and Fig. S4)—genera that typically include plant-associated bacteria. These multiple sequence alignments also identified the residue Glu213, establishing hydrophobic interactions with the indole moiety of IAA and IPA (Fig. 3B), as conserved in the same 13 enterobacterial strains (Fig. 6 and Fig. S3). To assess the individual contributions of these three residues in ligand coordination, mutants with site-directed mutations in Cys100, Glu213, and Cys215 were generated and auxin binding was investigated by isothermal titration calorimetry (ITC). These residues were mutated to the most common amino acids found at the equivalent positions in homologous proteins (Fig. 6 and Fig. S3) to create mutant substitutions C100S, E213Q, and C215Y. In accordance with the structural (Fig. 2 and Fig. 3) and MD simulation (Fig. 4 and Fig. S2A) data, we found that mutation of Cys215 abolished IAA and IPA binding (Fig. 7 and Table S1). Alternatively, mutation of Cys100 caused 3.4- and 4.9-fold decreases in the affinity for IAA and IPA, respectively (Fig. 7 and Table S1). Mutation of Glu213 did not significantly alter auxin binding by AdmX-LBD (Fig. 7 and Table S1).

10.1128/mbio.03363-22.3 FIG S3 Multiple-sequence alignment of AdmX-LBD with ligand binding domains (LBDs) of selected LysR transcriptional regulators. For the selection of LBDs, the AdmX-LBD sequence was used as a query for a BLAST search against the entire RefSeq protein database with the expected threshold set to 0.05. The alignment shows a selection of the 1,569 resulting sequences. Residues identified in the crystal structures that establish hydrogen bonds with the indole moiety (blue) and the auxin side chains (red) are highlighted. Amino acids that establish hydrophobic interactions are shown in yellow. Residues numbers refer to full-length AdmX of *S. plymuthica* (Protein GenBank accession no. KYQ97099.1). The mode by which the highlighted residues interact with IAA and IPA ligands is shown in Fig. 3. Ps, Providencia stuartii; Lc, *Lucilia cuprina*; Pg, Pluralibacter gergoviae; Ep, Erwinia persicina; Pa, Pantoea ananatis; Sp, Serratia plymuthica; Sm, Serratia marcescens; Se, *Serratia* sp.; Jc, *Jejubacter calystegiae*; Ec, Escherichia coli; Es, Escherichia sp.; Ef, Escherichia fergusonii; Ro, Raoultella ornithinolytica; Kar, Klebsiella aerogenes; Kv, Klebsiella variicola; Kq, Klebsiella quasipneumoniae; Ks, Klebsiella sp.; Kp, Klebsiella pneumoniae; Ka, Klebsiella *africana*. Download FIG S3, DOCX file, 0.02 MB. Copyright © 2023 Gavira et al. 2023 Gavira et al. https://creativecommons.org/licenses/by/4.0/ This content is distributed under the terms of the Creative Commons Attribution 4.0 International license.

10.1128/mbio.03363-22.4 FIG S4 Phylogenetic tree of the *Enterobacterales* subset. The genera with AdmX homologs containing the residues Cys100, Glu213, and Cys215 conserved are indicated in bold. The visualization is based on AnnoTree (K. Mendler, H. Chen, D. H. Parks, B. Lobb, L. A. Hug, et al., Nucleic Acids Res 47:4442–4448, 2019, https://doi.org/10.1093/nar/gkz246). Download FIG S4, JPG file, 0.8 MB. Copyright © 2023 Gavira et al. 2023 Gavira et al. https://creativecommons.org/licenses/by/4.0/ This content is distributed under the terms of the Creative Commons Attribution 4.0 International license.

The above results suggest that the presence of residues Cys100 and Cys215 may be a requisite to identify auxin sensing LTTRs. To validate this hypothesis, we selected three proteins based on taxonomy, host, and the conservation of key residues for auxin recognition for biochemical analyses. As expected, the LBD of protein WP_109886046.1 (AdmX_Kleb) from the human pathogen Klebsiella pneumoniae, which does not have conserved the key residues for auxin binding (Fig. 6 and Fig. S3), did not recognize IAA and IPA (Table S1), despite its high AdmX-LBD (identity, 79.3%; percentage positive, 86.8%). High-throughput screening based on differential scanning fluorimetry failed to identify signal molecules that are recognized by this protein. We subsequently analyzed the LBDs of the proteins WP_187509963.1 (AdmX_Erw) and WP_158151109.1 (AdmX_Pan) from the phytobacteria Erwinia persicina and Pantoea ananatis, respectively; two AdmX homologs that have the key residues for auxin binding. Unfortunately, both proteins were unstable under all experimental conditions tested, which prevented further biochemical analyses. To overcome this issue, we modeled the structures of the LBD of AdmX_Kleb, AdmX_Erw, and AdmX_Pan with AlphaFold 2 (35) using the structure of AdmX-LBD as the initial template. Subsequently, we conducted computational docking experiments with IAA and IPA. These analyses revealed good docking scores of AdmX_Erw-LBD and AdmX-LBD for both auxins, whereas slightly lower docking scores were obtained for AdmX_Pan. Consistent with our ITC data, no docking at the binding pocket was determined for AdmX-Kleb with IAA and IPA (Table 2) because the position equivalent to Cys215 at the binding site of AdmX is occupied by a tyrosine (Fig. 6) that prevents auxin access to the binding pocket. Taken together, our results strongly suggest that AdmX_Erw and AdmX_Pan bind auxins.

**TABLE 2:** Results from *in silico* docking binding studies of auxins to AdmX homologs

Protein	Docking SP score^*a*^	
	IAA	IPA
AdmX-LBD	−9.212	−9.830
AdmX_Erw-LBD	−7.582	−7.813
AdmX_Pan-LBD	−5.496	−6.070
AdmX_Kleb-LBD	No poses found	No poses found

a Shown are standard precision (SP) scores for the *in silico* docking of IAA and IPA to AdmX-LBD and to homology models of the LBDs of different AdmX homologs.

The limited number of AdmX homologs that have conserved residues Cys100 and Cys215 suggests a recent evolutionary emergence of these LTTRs. To investigate the evolutionary history of AdmX-LBD, we used a phylogenetic approach by constructing a maximum likelihood phylogenetic tree using the 1,560 sequences collected previously in the BLAST search. The results showed that AdmX-LBD forms a clade together with AdmX_Erw (WP_187509963.1) and AdmX_Pan (WP_158151109.1) from *E. persicina* and *P. ananatis*, respectively (Fig. 6). This phylogenetic clustering, together with the limited number of AdmX-LBD homologs that have the Cys100 and Cys215 residues conserved, suggest a recent evolutionary emergence of these LTTRs, specifically in plant-associated enterobacteria.

## DISCUSSION

Agonist compounds that bind to bacterial signal transduction receptors induce conformational changes that promote the activation of these systems (7, 9, 10). Alternatively, antagonists that bind to bacterial sensor domains to block agonist-induced responses have been identified for chemoreceptors (21–23), sensor kinases (19, 46), and transcriptional regulators, including LTTRs (24–26, 47). Although little is known about the reasons for the failure of antagonists to trigger a response, the limited data currently available suggest that these mechanisms are diverse (24, 26, 48, 49). Data also support the notion that the differences in the conformational changes induced by agonist and antagonist binding are minor. These small structural changes can be efficiently transmitted to the output domains to modulate the signaling response (e.g., transcriptional activity). Thus, although we showed here that IAA and IPA differ dramatically in their biological activities, their binding does not alter the oligomeric state of its receptor and only minor changes were observed in interdomain communication as monitored by DSC. The resolution of the 3D structures shows modest structural changes and alterations in the dimer interface. This is consistent with the notion that very minor differences in the structural changes induced by ligand binding determine whether a given ligand has agonistic or antagonistic effects.

Perhaps one of the best-characterized systems that is modulated by agonists and antagonists is the two-component system TodS/TodT, which regulates expression of the *tod* catabolic genes to control degradation of aromatic hydrocarbons in response of various substrates and nonsubstrates of the pathway (50). The sensor kinase TodS recognizes at its N-terminal PAS domain several agonist and antagonist signals (19). Agonists and antagonists compete for binding to TodS *in vitro*, and the presence of antagonists reduces the magnitude of the regulatory response mediated by agonists *in vivo* (19). Agonists and antagonists bind with similar affinities to TodS. Whereas agonists increase TodS autophosphorylation, antagonists had no significant effect (19). Our results showed a number of parallels between TodS and AdmX. (i) Like IAA and IPA, the TodS agonists and antagonists are structurally very similar. For example, methylbenzene (toluene) is the most potent agonist, whereas 1,2,4-trimethylbenzene is an antagonist (19). (ii) Similar to IAA and IPA, the binding modes and orientations of toluene and 1,2,4-trimethylbenzene in the biding pocket of the TodS PAS sensor domain are highly similar (48). (iii) As for the IPA- and IAA-complexed AdmX-LBD structures, the most noticeable differences in the PAS structures bound to toluene and 1,2,4-trimethylbenzene are subtle changes in the secondary well-defined structural regions (48). In their model, the authors propose that toluene binding strengthens the dimeric state of the autokinase domain, causing an increase in autokinase activity. In this regard, we found that IPA binding to AdmX-LBD causes a higher degree of compactness of the AdmX-LBD dimer, resulting in a smaller total surface area of the dimer. This increased AdmX-LBD packing when bound to IPA may result in a reduced conformational flexibility of the protein, which is of relevance for the correct regulatory function of LTTRs (7, 9, 10). Analogously, crystal structures of the LBD of the LTTR PqsR in complex with the agonist 2-nonyl-4-hydroxyquinoline and the antagonist quinazolinone also revealed important similarities to AdmX, including subtle conformational changes in the structure of PqsR-LBD in the presence of agonists and antagonists (26). Another representative example is the TR CviR, as only minor structural differences (e.g., RSMD of 0.8 Å) were measured for a superimposition of CviR-LBD of bound to agonists and antagonists (24). Nonetheless, antagonist binding to full-length CviR caused strong structural changes in the DBD that resulted in a conformation that was unable to bind to the target DNA (24). In this regard, calorimetric analyses revealed that the thermal stability of AdmX-LBD in the presence of IAA and IPA was comparable to that of the full-length protein (29). However, IAA and IPA modulated in a slightly different manner the unfolding characteristics of the DBD, causing shifts in *T_m_* by 1.7 and 1.2°C, respectively, and a higher calorimetric unfolding enthalpy change was measured in the DBD upon IAA binding (Fig. 1C and see Table S1 in the supplemental material). We hypothesize that these differences are due to a dissimilar interdomain signal transmission in AdmX in response to IAA and IPA. In accordance, IAA and IPA were shown to cause different changes to the secondary structure content of AdmX upon binding (29).

In general, LBDs are rapidly evolving domains (51, 52). This is reflected by a contrast between the high sequence conservation of the signaling domains of chemoreceptors (53) and a high degree of diversity of their sensor domains (54). For example, a recent study revealed that sensor domains of paralogous chemoreceptors evolve rapidly through changes in individual residues that ultimately result in alterations of the ligand spectrum (52). In this regard, multiple orthologs and paralogs of LTTRs are found in bacterial genomes (7, 8). Analogously, whereas DBDs of LTTRs are highly conserved in sequence, high diversity at the amino acid level is observed in their LBDs (7, 8)—as a strong indication that sensor domains of LTTRs are subject to high selective pressures that drive their functional specialization. Indeed, there is increasing experimental evidence supporting the idea that ecological factors play an important role in the evolution of bacterial sensor domains. For example, the human pathogen Pseudomonas aeruginosa has evolved three dCache containing chemoreceptors that recognize different human neurotransmitters (52, 55, 56). In addition, phylogenomic analyses showed that plant-associated bacteria are enriched in receptors with specific LBD types, and this specificity in the sensor domain types was found to be independent of phylogeny but rather linked to the lifestyle in association with plant hosts (57). The combination of approaches based on structural biology, phylogenetics, computational biology, and protein biochemistry has allowed us to advance in the study of the evolutionary history of the LBD of AdmX. While amino acid residues involved in the coordination of the indole moiety are conserved in homologous proteins of bacteria isolated from different environmental sources and hosts, those residues that establish interactions with the side chains of IAA and IPA are conserved in plant-associated bacteria of the closely related *Serratia*, *Pantoea*, and *Erwinia* genera (Fig. 6 and Fig. S3), which is indicative that these proteins may bind auxins. Given the narrow phyletic distribution and the recent evolutionary history of AdmX homologs, their emergence is likely to be the result of plant-driven evolutionary pressures, where amino acid substitutions confer on the proteins the capacity to sense auxin phytohormones. This notion is consistent with *in vitro* accelerated evolution experiments that have increased the specificity of the TrpR regulator toward IAA through the mutation of key residues in the ligand binding pocket, resulting in an about 100-fold increase in auxin affinity (58).

The phytohormone IAA is the main naturally occurring auxin, which regulates plant growth, development, and defense against abiotic and biotic stresses, playing essential roles in cell division, flowering, organogenesis, seed and root development, among other processes (30–32, 59). IAA production is ubiquitous in all kingdoms of life (60–64) and modulates a wide diversity of biological processes, including inflammatory processes in humans (65), microalgal growth (66), fungal physiology, sporulation, and filamentous growth (64) as well as bacterial physiology, metabolism, and virulence (29, 60, 67, 68). The broad phyletic distribution of organisms able to synthesize IAA, together with the diversity of processes that modulates, has converted this auxin into a pivotal signal molecule for intra- and interkingdom communication research (29, 60, 69–71). However, outside the field of plant biology, knowledge on the molecular mechanisms by which IAA modulates this broad diversity of biological functions is still scarce. Indeed, independently of AdmX, a very limited number of IAA sensor proteins have been identified in bacteria, including the chemoreceptor PcpI of Pseudomonas putida (71), the transcriptional regulator TrpR of Escherichia coli (58), and several MarR bacterial regulators of auxin catabolism (72) (Fig. S5). Next to their role in transport, solute binding proteins play an important role in signal transduction and the solute binding protein Dde_0634 of Oleidesulfovibrio alaskensis G20 was also shown to bind IAA (73) (Fig. S5). Our data thus contribute to a deeper understanding of the molecular mechanisms of auxin recognition by bacteria. IAA binding by AdmX homologs may allow a rapid and efficient bacterial adaptation to different plant hosts and plant-associated environments (e.g., the rhizosphere), where IAA can be found at micromolar concentrations (74, 75).

10.1128/mbio.03363-22.5 FIG S5 Three-dimensional structures of bacterial sensor proteins and solute binding proteins that bind indole-3-acetic acid. AdmX-LBD (PDB ID 7QEJ) (A), IacR (PDB ID 7KUA) (B), TrpR (PDB ID 6EJW) (C), and the solute binding protein Dde_0634 (PDB ID 4PGP) (D) are shown. IAA is shown in sphere mode. Download FIG S5, JPG file, 0.6 MB. Copyright © 2023 Gavira et al. 2023 Gavira et al. https://creativecommons.org/licenses/by/4.0/ This content is distributed under the terms of the Creative Commons Attribution 4.0 International license.

Taken together, our study highlights the plasticity of the molecular mechanisms by which TRs modulate gene expression in response to environmental signals and reinforces the power of structural biology and computational biology for deciphering the agonist- and antagonist-induced conformational changes. Further research is needed to understand the physiological role of antagonist perception by bacterial sensing proteins. Natural antagonists have been reported to function as modulators of cellular responses under stress conditions in plants and algae (76, 77), regulators of fungal metabolism, growth, and development (78), and inhibitors of conjugative processes in bacteria (79). In addition, signal antagonists may function as inter- and intrakingdom signaling molecules modulating different physiological and metabolic processes in bacteria (24, 25, 80). These dual-signaling mechanisms may thus be common in nature, allowing to confer fitness advantages to (micro)organisms under conditions of opposing selective pressures.

## MATERIALS AND METHODS

### Culture conditions, bacterial strains and plasmids.

The strains and plasmids used in this study are listed in Table S5 in the supplemental material. E. coli strains were routinely grown at 37°C in LB medium. When required, kanamycin was used at 50 μg/mL.

10.1128/mbio.03363-22.10 TABLE S5 Strains and plasmids used in this study. Download Table S5, DOCX file, 0.02 MB. Copyright © 2023 Gavira et al. 2023 Gavira et al. https://creativecommons.org/licenses/by/4.0/ This content is distributed under the terms of the Creative Commons Attribution 4.0 International license.

### Protein expression purification.

AdmX, AdmX-LBD, and WP_109886046.1-LBD were expressed in Escherichia coli BL21-AI (AdmX and AdmX-LBD) and E. coli BL21(DE3) (WP_109886046.1-LBD), and purified by metal affinity chromatography, as described previously (29). Mutant proteins were purified following the protocol for the wild-type protein. AdmX-LBD comprises amino acids 69 to 295 of AdmX (GenBank accession no. KYQ97099). Analytical ultracentrifugation (AUC), differential scanning calorimetry (DSC), and isothermal titration calorimetry (ITC) experiments were conducted in mixtures of 50 mM KH_2_PO_4_–K_2_HPO_4_, 300 mM NaCl, 10% (vol/vol) glycerol, and 2 mM β-mercaptoethanol (pH 7.0) for AdmX and 20 mM HEPES, 150 mM NaCl, 2 mM β-mercaptoethanol (pH 7.4) for AdmX-LBD, AdmX-LBD mutant variants, and WP_109886046.1-LBD.

### Isothermal titration calorimetry.

Measurements were made using a VP-ITC titration calorimeter (Microcal, Inc., Northampton, MA, USA) at 30°C following the protocol previously published (29). Proteins at 50 to 100 μM were titrated with 6.4- to 9.6-μL aliquots of 1 to 5 mM indole-3-acetic acid (IAA) and indole-3-pyruvic acid (IPA). The mean enthalpies measured from the injection of ligands into the buffer were subtracted from raw data prior to data fitting using the “One binding site model” of the MicroCal version of the ORIGIN software.

### Differential scanning fluorimetry-based high-throughput ligand screening.

Thermal shift assays were performed on a MyIQ2 real-time PCR instrument (Bio-Rad), as previously described (29). Ligands from the PM1, PM2A, PM3B, PM4A, and PM5 compound arrays (Biolog, Hayward, CA, USA) were dissolved in 50 μL of Milli-Q water, which, according to the manufacturer, corresponds to a concentration of 10 to 20 mM.

### Analytical ultracentrifugation.

Analytical ultracentrifugation (AUC) experiments were conducted with a ProteomeLab XL-I (Beckman-Coulter, Palo Alto, CA) equipped with interference and absorbance optics. Sedimentation was carried out at 42,000 rpm and 10°C in an eight-hole Ti-50 Beckman Coulter rotor and monitored with absorbance optics at 280 nm in continuous mode. Recombinant AdmX at 27 μM and its individual ligand binding domain (AdmX-LBD) at 58 μM were measured in the absence and presence of either IAA (1 mM) or IPA (150 μM) dialyzed in the buffers described above. Both IPA and IAA absorb at 280 nm, interfering with protein absorbance. Therefore, both AUC absorbance and interference optics were used simultaneously, and the results from interference were chosen in the cases where absorbance data could not be analyzed correctly. Dialysis buffers with the corresponding ligand were used as the reference buffer. Density (1.045 g/mL for AdmX and 1.0076 for AdmX-LBD) and viscosity (1.854 cP for AdmX and 1.344 cP for AdmX-LBD) of the buffers were calculated with SEDNTERP (81). Sedimentation data analysis was performed with Sedfit v.15.01b from reference 82, using the noninteracting discrete species continuous Svedberg distribution model [*c*(*s*)]. After the fitting procedure, data were exported to GUSSI v.1.3.2 (83) for figure preparation. To assign the sedimentation coefficient observed to the corresponding oligomeric species, theoretical *s* values were calculated via HYDROPRO (36) hydrodynamic modeling software, applied to the structures of AdmX and AdmX-LBD as modeled by AlphaFold 2 (35), with a 2.9-Å radius for the primary elements. Conversion from experimental sedimentation values (S) to standard sedimentation coefficients (*s*_20,_*_w_*) was done using the equation *s*_20,_*_w_* = *s*_exp_ (η_exp_/η_20,_*_w_*) (1 − *ṽ*ρ_20,_*_w_*/1 − *ṽ*ρ_exp_), as described previously (84), with η representing the density, ρ the solvent density and *ṽ* the protein partial-specific volume.

### Differential scanning calorimetry.

Differential scanning calorimetry (DSC) experiments were conducted with a MicroCal DSC-PEAQ system (Malvern Panalytical, United Kingdom) at a scan rate of 90°C/h from 10 to 85°C. AdmX and AdmX-LBD in the absence and presence of either IPA or IAA were measured. Calorimetric cells were kept under pressure (60 lb/in^2^) to prevent sample degassing. Buffer-buffer (including the ligand-containing buffers) baselines obtained after each protein scan were subtracted from the signal and the reversibility of protein unfolding was investigated by rescanning twice. AdmX at 27 μM and AdmX-LBD at 58 μM were measured in the absence and presence of IAA or IPA at a 1 mM concentration in the dialysis buffers. The calorimetric enthalpies were estimated by integration of the transition peaks after subtracting the buffer-buffer baselines, using the non-two-state model of the MicroCal DSC-PEAQ and Origin software. Analysis of the curves was smoothed with the Savitzky-Golay algorithm from raw data.

### Crystallization, data collection, structure determination, and analysis.

AdmX-LBD in a mixture of 20 mM Tris-HCl, 150 mM NaCl, and 2 mM β-mercaptoethanol (pH 7.4) was concentrated to 15 mg/mL using 10-kDa-cutoff Centricon concentrators (Amicon). Prior to concentration, IAA or IPA was added to the protein at a final concentration of 10 mM. The excess ligand was removed by centrifugation buffer exchange using 10-kDa-cutoff filters (Amicon). The resulting protein was used for initial crystallization screening by both the hanging-drop vapor diffusion (HDvd) and the capillary counterdiffusion (Ccd) techniques (85). HDvd experiments were set up in 24-well VDX crystallization plates (Hampton Research) using Hampton Research screen I, with droplets prepared by mixing protein solution with reservoir solution in a 1:1 ratio that were then equilibrated against 500 μL reservoir solution. Ccd experiments were set up by loading the apo- and holo-AdmX-LBDs into 0.2-mm-inner-diameter capillaries that were then equilibrated against an excess of precipitant cocktails prepared *ad hoc* (86). The crystallization setups were kept at 293 K and inspected regularly. AdmX-LBD crystallized in complex with IAA or IPA. Prior to data collection, crystals were equilibrated in mother solution supplemented with 20% to 30% (vol/vol) polyethylene gloycol 200 (PEG 200) or directly looped out with the help of individual LithoLoops (Molecular Dimensions) and flash-cooled in liquid nitrogen for storage.

Data were collected at beamlines ID23-1, ID23-2, and ID30A-3 of the European Synchrotron Radiation Facility (ESRF; Grenoble, France) and at the Xaloc beamline of the Alba synchrotron radiation source (Barcelona, Spain). Data were indexed and integrated with XDS (87), then scaled and reduced with AIMLESS (88) of the CCP4 program suite (89). Crystals diffracted to a resolution of around 2.0 Å, but efforts to phase the data failed, including crystal improvement and polymorph search by removing the His tag and by producing Se-Met derivatives. The phase problem was solved using a model of AdmX-LBD generated by AlphaFold 2 (35) to feed ARCIMBOLDO_SHREDDER (90). ARCIMBOLDO provided a polyalanine model consisting of 261 residues. Refinement was initiated with phenix.refine (91) of the PHENIX suite (92) and Refmac (93) of the CCP4 program suite (89). After manual model building, water inspection and ligand identification were done in Coot (94) and final refinement was assessed, including titration-libration-screw (TLS) parameterization (95). Both models were verified with Molprobity (96) and the PDB validation server prior to being deposited at the Protein Data Bank in Europe (PDBe). The data collection and refinement statistics are provided in Table S2.

### Molecular dynamics simulations and analysis.

All stages of protein modeling, molecular dynamics (MD), and analytical calculations were performed using the Schrödinger molecular modeling suite v.2021-1 (Schrödinger, LLC, New York, NY, USA). MD simulations were performed using the Desmond package (97). The MD system was set-up in the Maestro’s “System Builder” utility as follows. A TIP3P water model (98) was used to simulate water molecules: the buffer distance in the orthorhombic box was set at 10 Å, the recalculated amount of Na^+^/Cl^−^ ions was added to balance the system charge, and the ions were placed randomly to neutralize the solvated system. Additional salt was appended to a final concentration of 0.15 M in order to simulate physiological conditions. MD simulations were conducted with the periodic boundary conditions in the isothermal-isobaric ensemble class using OPLS4 force field parameters (99). The temperature and pressure were kept at 300 K and 1 atmospheric pressure, respectively, using Nosé-Hoover temperature coupling and isotropic scaling (100). The model system was relaxed before simulations using Maestro's default relaxation protocol, including two stages of minimization (restrained and unrestrained) followed by four stages of MD runs with gradually diminishing restraints. MD simulations were carried out by running the 100- and 500-ns recording trajectory configurations obtained at 50-ps intervals. First, a 100-ns MD run was used for a complex relaxation and stabilization where the resulting MD trajectories were clustered by RMSD. The structure with minimal free energy was chosen for subsequent 500-ns analytical MD simulation. RMSD clustering was performed using the Desmond package (97).

The MD trajectory files were investigated using simulation quality analysis (SQA) and simulation interaction diagram (SID) programs available within the Desmond module. SID was employed to generate the protein and ligand root mean square fluctuations (RMSF) and root mean square deviation (RMSD), ligand interaction fingerprints and interaction fractions, and secondary structure element (SSE) dynamics of the protein. The retrieved values were then plotted using the R package v.4.2.1. The Prime module in Schrödinger suite 2021-1 was used to compute the ligand binding energies through the use of a physics-based MM/GBSA method (101, 102). The OPLS4 force field and VSGB solvation model were used in the calculations of ligand binding.

### Phylogenetic analysis.

The sequence of AdmX-LBD was used it as a query for a BLAST search against the RefSeq protein database (103) with the expect threshold set to 0.05. All 1,569 resulting sequences were aligned using MAFFT (104). The resulting multiple sequence alignment was subjected to maximum likelihood phylogenetic tree construction using iqTree (105).

### Molecular docking.

Structural models were generated with AlphaFold 2 (35) using default parameters and feed with the structure of AdmX-LBD as the template. All targets were processed with the Protein Preparation Wizard in the Schrödinger suite (106). Hydrogen atoms were added followed by the adjustment of bond orders. The protonation and tautomeric states for protonable residues were adjusted to match pH = 7.0. Water molecules with less than 3 H-bonds to the active site were deleted. Proteins were finally subjected to geometry optimization by using OPLS_3 force field (107).

Docking tests were performed using the software Glide (108). All grid boxes for molecular docking were centered in the approximate ligand pocket extracted from AdmX-LBD crystal structure. The grid boxes’ dimensions were tested at 10 by 10 by 10 Å in order to include all binding sites. Standard precision (SP) and extra precision (XP) Glide modes were proved.

### Data availability.

Coordinates and the experimental structure factors have been deposited in the Protein Data Bank (PDB) with identifiers 7QEJ (AdmX-LBD/IAA) and 7QEK (AdmX-LBD/IPA).
