# Supplementary material for: Emergence of an Auxin Sensing Domain in Plant-Associated Bacteria
Source: mBio. 2023 Jan 5;14(1):e03363-22. doi: 10.1128/mbio.03363-22 (PMC9973260; doi:10.1128/mbio.03363-22)
Supplement: TABLE S1 [file mbio.03363-22-s0006.docx]

**Table S1. Thermodynamic parameters derived from differential scanning calorimetry and microcalorimetric titrations.**

| Differential scanning calorimetry | | | | |
| --- | --- | --- | --- | --- |
| Protein (and ligand) | **DNA binding domain (DBD)** | | **Ligand binding domain (LBD)** | |
|  | **T_1_ (ºC)** | **∆*H*_1_ (kJ/mol)** | **T_2_ (ºC)** | **∆*H*_2_ (kJ/mol)** |
| AdmX | 45.54 | 8.89 | 59.34 | 75.7 |
| AdmX + IAA | 47.23 | 11.85 | 64.01 | 76.7 |
| AdmX + IPA | 46.73 | 10.40 | 68.61 | 81.62 |
| AdmX-LBD | - | - | 59.08 | 243.11 |
| AdmX-LBD + IAA | - | - | 63.93 | 279.80 |
| AdmX-LBD + IPA | - | - | 69.01 | 308.43 |

| **Isothermal titration calorimetry** | | | | |
| --- | --- | --- | --- | --- |
| **Protein** | **Organism** | **Compound** | ***K*_D_ (µM)** | **Δ*H* (kcal/mol)** |
| AdmX-LBD | *Serratia plymuthica* A153 | Indole-3-acetic acid | 15.2 ± 2.0^a^ | -0.54 ± 0.1^a^ |
|  |  | Indole-3-pyruvic acid | 6.4 ± 0.2^a^ | -9.8 ± 0.2^a^ |
| AdmX-LBD_C100S | *Serratia plymuthica* A153 | Indole-3-acetic acid | 51.0 ± 3.4 | -5.0 ± 0.5 |
|  |  | Indole-3-pyruvic acid | 31.5 ± 1.6 | -24.6 ± 1.7 |
| AdmX-LBD_E213Q | *Serratia plymuthica* A153 | Indole-3-acetic acid | 23.9 ± 2.4 | -4.0 ± 0.6 |
|  |  | Indole-3-pyruvic acid | 6.0 ± 0.7 | -10.2 ± 0.6 |
| AdmX-LBD_C215Y | *Serratia plymuthica* A153 | Indole-3-acetic acid | No binding | |
|  |  | Indole-3-pyruvic acid | No binding | |
| WP_109886046.1^b^-LBD (AdmX_Kleb-LBD) | *Klebsiella pneumoniae* | Indole-3-acetic acid | No binding | |
|  |  | Indole-3-pyruvic acid | No binding | |
| WP_158151109.1^b^-LBD (AdmX_Pan-LBD) | *Pantoea ananatis* | Protein unstable | | |
| WP_187509963.1^b^-LBD(AdmX_Erw-LBD) | *Erwinia* *persicina* | Protein unstable | | |

^a^Data obtained from (M.A. Matilla, A. Daddaoua, A. Chini, B. Morel, and T. Krell, Nucl Acids Res 46: 11229-11238, doi: 10.1093/nar/gky766).

^b^NCBI Protein identity.
