# Supplementary figures and images for: Emergence of an Auxin Sensing Domain in Plant-Associated Bacteria
Source: mBio. 2023 Jan 5;14(1):e03363-22. doi: 10.1128/mbio.03363-22 (PMC9973260; doi:10.1128/mbio.03363-22)

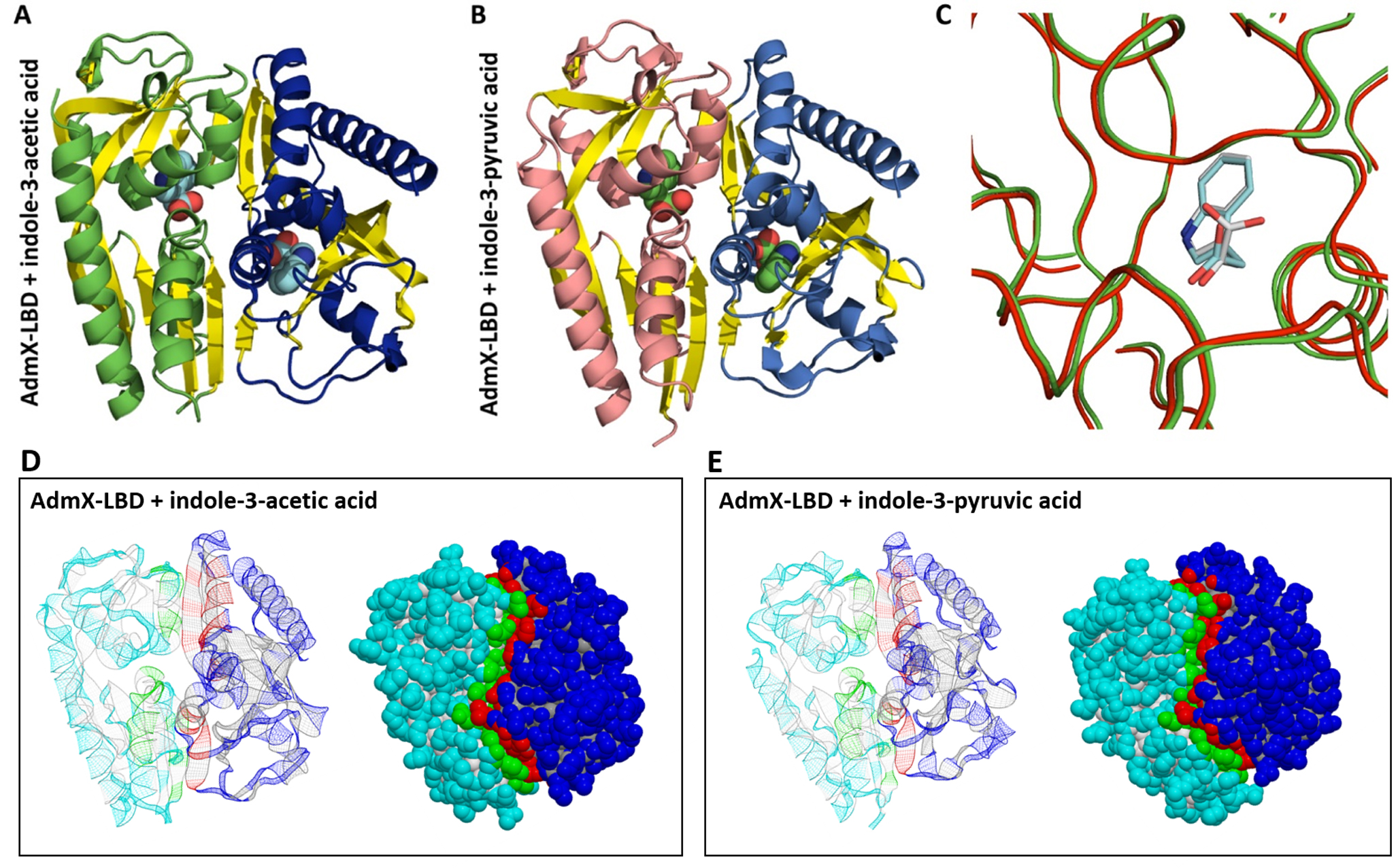

Supplement: FIG S1 [file mbio.03363-22-s0001.jpg]

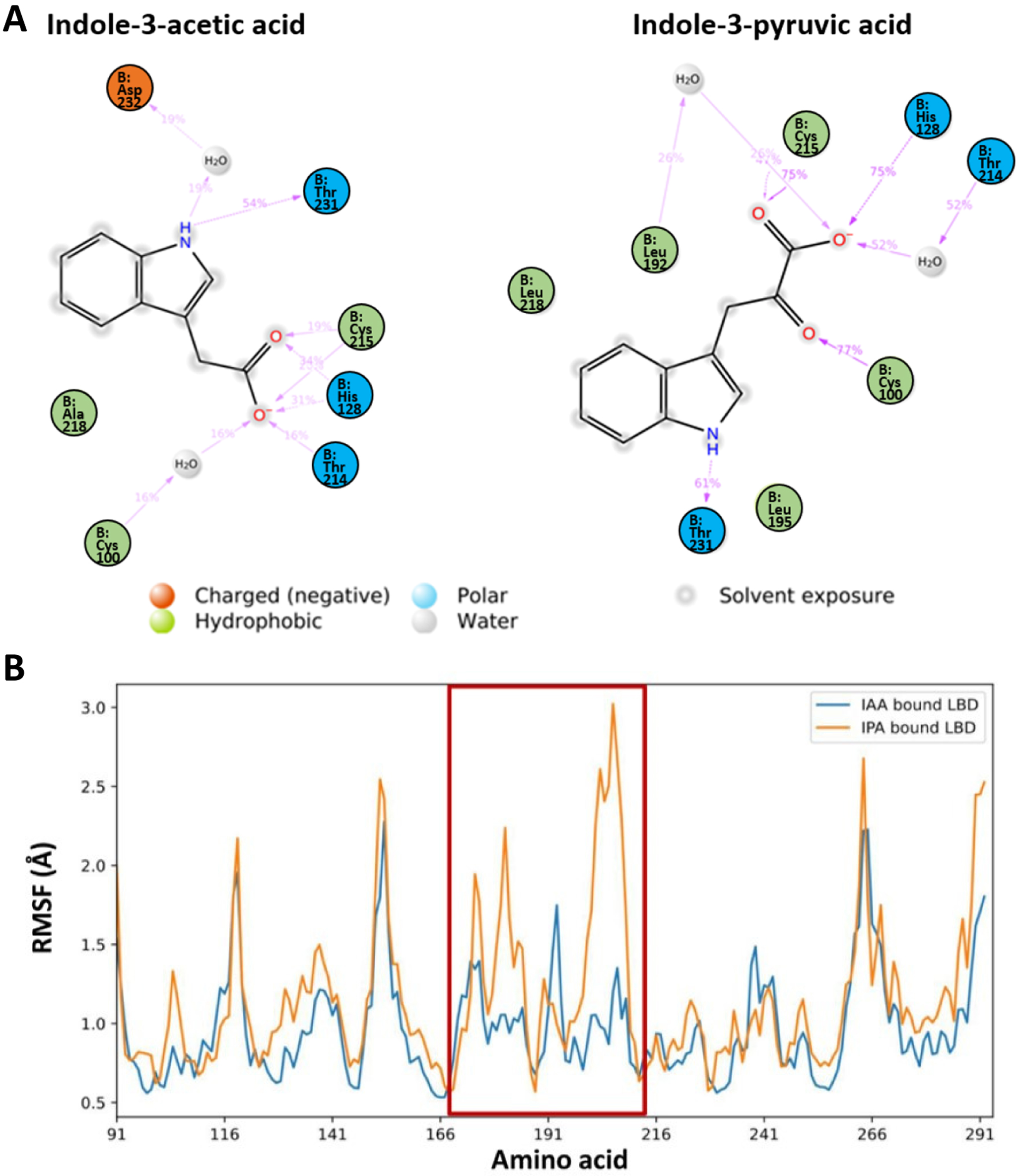

Supplement: FIG S2 [file mbio.03363-22-s0002.jpg]

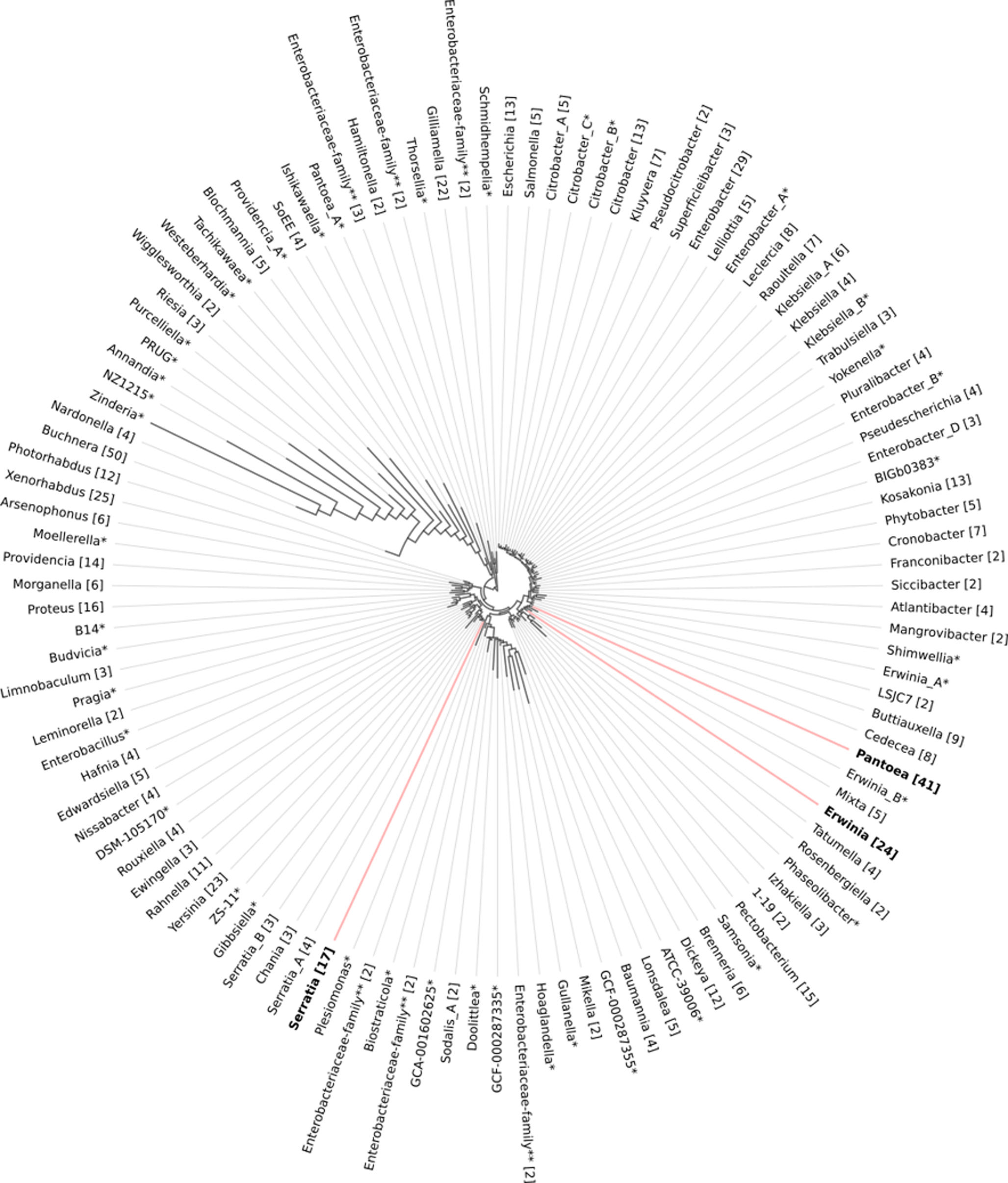

Supplement: FIG S4 [file mbio.03363-22-s0004.jpg]

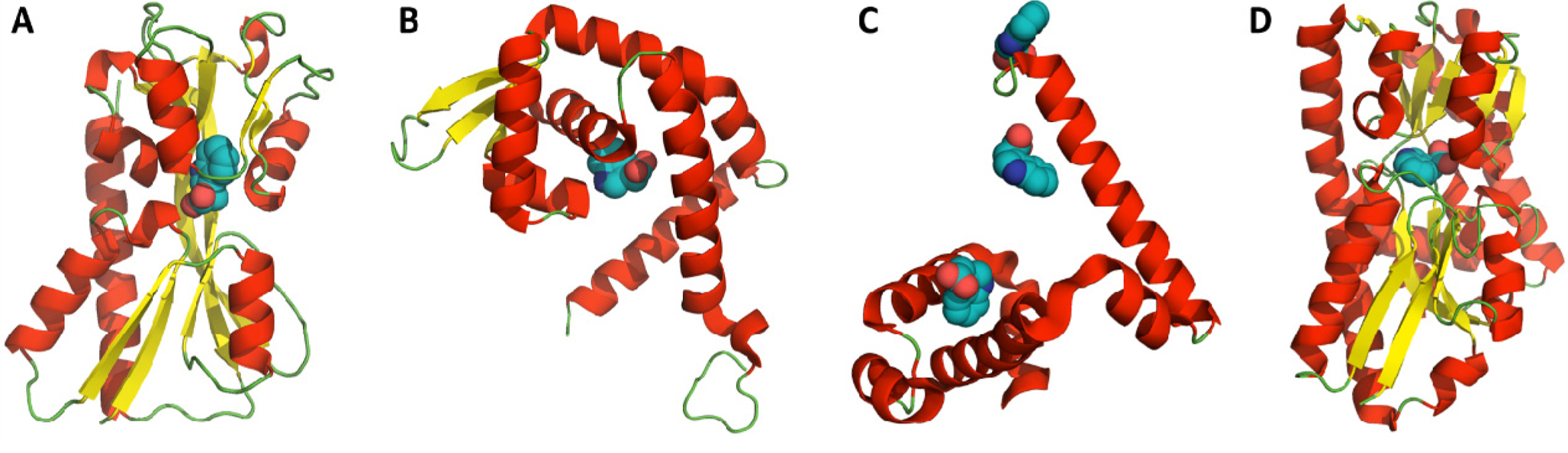

Supplement: FIG S5 [file mbio.03363-22-s0005.jpg]
