# Supplementary material for: Emergence of an Auxin Sensing Domain in Plant-Associated Bacteria
Source: mBio. 2023 Jan 5;14(1):e03363-22. doi: 10.1128/mbio.03363-22 (PMC9973260; doi:10.1128/mbio.03363-22)
Supplement: TABLE S2 [file mbio.03363-22-s0007.docx]

## **Table S2. Data collection and refinement statistics of different structures of AdmX-LBD.** Statistics for the highest-resolution shell are shown in parentheses.

| **Ligand** | **IAA** | **IPA** |
| --- | --- | --- |
| PDB ID. | 7QEJ | 7QEK |
| Beam Line | XALOC (ALBA) | MASSIF-3 (ESRF) |
| Space group | P 2_1_ 2_1_ 2 | P 2_1_ 2_1_ 2_1_ |
| Unit cell a, b, c (Å) | 87.12, 92.14, 49.92 | 53.21, 76.04, 94.134 |
| Resolution (Å) | 63.31 - 1.81 (1.875 - 1.81) | 35.26 - 2.25 (2.33 - 2.25) |
| Unique reflections | 36996 (3667) | 18464 (1838) |
| Multiplicity | 4.2 (4.3) | 4.5 (4.7) |
| Completeness (%) | 98.85 (99.57) | 98.43 (99.24) |
| I/σ_I_ | 11.79 (1.43) | 16.37 (1.54) |
| Wilson B-factor | 30.58 | 49.44 |
| R*_merge_* (%) | 6.44 (80.61) | 5.50 (88.21) |
| CC(1/2) | 0.998 (0.643) | 0.999 (0.629) |
| **Refinement** | | |
| R*_work_*/R*_free_* (%) | 18.00 / 21.02 | 20.74 / 25.76 |
| No. atoms | 3587 | 3433 |
| Protein | 3417 | 3372 |
| Ligands | 44 | 31 |
| Solvent | 142 | 30 |
| B-factor (Å^2^) | 41.55 | 60.91 |
| R.m.s deviations |  |  |
| Bond lengths (Å) | 0.017 | 0.004 |
| Bond angles (°) | 1.36 | 0.79 |
| Ramachandran (%) |  |  |
| Favored (%) | 98.26 | 96.52 |
| Outliers (%) | 0.00 | 0.00 |
