# Supplementary material for: Emergence of an Auxin Sensing Domain in Plant-Associated Bacteria
Source: mBio. 2023 Jan 5;14(1):e03363-22. doi: 10.1128/mbio.03363-22 (PMC9973260; doi:10.1128/mbio.03363-22)
Supplement: TABLE S3 [file mbio.03363-22-s0008.docx]

**Table S3. Root-mean-square displacement (RMSD) values (in Å) for Cα atoms of individual chains AdmX-LBD in complex with IAA and IPA.**

|  | | **IAA** | | **IPA** | |
| --- | --- | --- | --- | --- | --- |
| **Ligand** | **Chain** | **A** | **B** | **A** | **B** |
| **IAA** | **A** | - | 0.780 | 0.759 | 0.853 |
|  | **B** |  | - | 0.786 | 0.777 |
| **IPA** | **A** |  |  | - | 0.504 |
|  | **B** |  |  |  | - |
