# Supplementary material for: Emergence of an Auxin Sensing Domain in Plant-Associated Bacteria
Source: mBio. 2023 Jan 5;14(1):e03363-22. doi: 10.1128/mbio.03363-22 (PMC9973260; doi:10.1128/mbio.03363-22)
Supplement: TABLE S4 [file mbio.03363-22-s0009.docx]

**Table S4. Analysis of the AdmX-LBD dimer interface statistics obtained from PDBsum and PISA.**

| **PDBsum^a^** | | | | | | | | | |
| --- | --- | --- | --- | --- | --- | --- | --- | --- | --- |
| **Protein (ligand)** | | **Chain** | | | **Interface area (Å^2^)** | | **No. of salt bridges** | | **No. of hydrogen bonds** |
| **AdmX-LBD (IAA)** | | A | | | 1511 | | 2 | | 12 |
|  |  | B | | | 1514 | |  |  |  |
| **AdmX-LBD (IPA)** | | A | | | 1560 | | 6 | | 12 |
|  |  | B | | | 1565 | |  |  |  |
| **PISA^b^** | | | | | | | | | |
| **Protein (ligand)** | **Chain** | | **Surface area (Å^2^)** | **Buried Area (Å^2^)** | | **ΔG^int^ (kcal/mol)**^c^ | | **Total number of interactions (No. of salt bridges + No. of hydrogen bonds)** | |
| **AdmX-LBD (IAA)** | AB | | 17200 | 1561.3 | | -13.7 | | 14 | |
|  | AB(IAA)_2_ | |  | 4120 | | -11.6 | |  |  |
| **AdmX-LBD (IPA)** | AB | | 17760 | 1544.9 | | -17.1 | | 32 | |
|  | AB(IPA)_2_ | |  | 4080 | | -24.6 | |  |  |

^a^Laskowski RA, Jabłońska J, Pravda L, Vařeková RS, Thornton JM. 2018. PDBsum: Structural summaries of PDB entries. Protein Sci 27:129–134.

^b^Krissinel E, Henrick K. 2007. Inference of macromolecular assemblies from crystalline state. J Mol Biol 372:774–797.

^c^ΔG^int^: indicates the solvation free energy gain upon dimer. This value does not include the effect of satisfied hydrogen bonds and salt bridges across the assembly's interfaces.
