# Supplementary material for: Emergence of an Auxin Sensing Domain in Plant-Associated Bacteria
Source: mBio. 2023 Jan 5;14(1):e03363-22. doi: 10.1128/mbio.03363-22 (PMC9973260; doi:10.1128/mbio.03363-22)
Supplement: FIG S3 [file mbio.03363-22-s0003.docx]

**C100 H128**

**KYQ97099.1_(AdmX)** QGIYQDLSNLRLLADNLARDPRAKFTLGCLPCLGLSLVPEIATDFYQQNSNLVMTLTAEH

WP_070926383.1_(Ps) KEVYRNLDRLRILAQNLSKNPQGKLAIGCLPSLGLNLVPEVTAQFIKKHPNIKLTIGTHH

XP_023295947.1_(Lc) KEVYRNLDRLRILAQNLSKNPQGKLAIGCLPSLGLNLVPEVTAQFIKKHPNIKLTIGTHH

WP_043083564.1_(Pg) QHIYQDLDSLRLLADNLARDPRARLSLGCLPSLGLSLVPGIVTDFYQQNANLVMTLSTEH

**WP_187509963.1_(Ep)** QSIYQDLDNLRLLADNLARDPRAKFTLGCLPCLGLSLVPELATDFYQQNSNMVMTLTTEH

**WP_158151109.1_(Pa)** QSIYQELDNLRLLADNLARDPRAKFTLGCLPCLGLSLVPELASDFYQQNSNLVMTLTTEH

**WP_116219602.1_(Pa)** QSIYQDLDNLRLLADNLARDPRAKFTLGCLPCLGLSLVPELASDFYQQNSNLVMTLTTEH

**WP_062867893.1_(Sp)** QGIYQDLSNLRLLADNLARDPRAKFTLGCLPCLGLSLVPEIATDFYQQNSNLVMTLTAEH

**WP_126528367.1_(Sp)** QGIYQDLSNLRLLADNLARDPRAKFTLGCLPCLGLSLVPEIATDFYQQNNNLVMTLTTEH

**WP_126483812.1_(Sp)** QGIYQDLSNLRLLADNLARDPRAKFTLGCLPCLGLSLVPEIATDFYQQNNNLVMTLTTEH

**WP_062790196.1_(Sp)** QGIYQDLSNLRLLADNLARDPRAKFTLGCLPCLGLSLVPEIATDFYQQNSNLVMTLTTEH

**WP_212558166.1_(Sp)** QGIYQDLSNLRLLADNLARDPRAKFTLGCLPCLGLSLVPEIATDFYQQNSNLVMTLTTEH

**WP_219227858.1_(Sm)** QGIYQDLSNLRLLADNLARDPRAKFTLGCLPCLGLSLIPEIATDFYQQNSNLEMTLTTEH

**WP_103773490.1_(Sm)** QGIYQDLSNLRLLADNLARDPRAKFTLGCLPCLGLSLIPEIATDFYQQNSNLEMTLTTEH

**WP_086580121.1_(Sm)** QGIYQDLSNLRLLADNLARDPRAKFTLGCLPCLGLSLIPEIATDFYQQNSNLEMTLTTEH

**WP_073532156.1_(Sm)** QGIYQDLSNLRLLADNLARDPRAKFTLGCLPCLGLSLVPEIATDFYQQNSNLVMTLTTEH

**WP_046896471.1_(Se)** QGIYQDLSNLRLLADNLARDPRAKFTLGCLPCLGLSLVPEIATDFYQQNSNLEMTLTTEH

WP_138096251.1_(Jc) QTVYRDLDNLRLLADNLARDPRAKIALGCLPSLGLSLVPGIVTDFYQQNANLVMTLSTDH

WP_196611696.1_(Ec) QSIYHELDNLRVLADNLTRDPRARIALGCLPSLGLSLVPEIVTAFYQQNANLVMTLTTEH

WP_000776564.1_(Es) QSIYHELDNLRLLADNLTRDPRARIALGCLPSLGLSLVPEIVTAFYQQNANLVMTLTTEH

WP_196081520.1_(Ef) QSIYHELDNLRLLADNLTRDPRARIALGCLPSLGLSLVPEIVTAFYQQNANLVMTLTTEH

WP_216711806.1_(Ro) QNIYQDLDNLRLLADNLARDPRAKMSLGCLPSLGLSLVPELVTDFYQQNANLVMTLTTEH

WP_143958904.1_(Ro) QNIYQDLDNLRLLADNLARDPRAKMSLGCLPSLGLSLVPELVTDFYQQNANLVMTLTTEH

WP_032714315.1_(Kar) QSIYQDLDNLRLLADNLARDPRAKITLGCLPSLGLSLVPELVTDFYQQNSNLVMTLTTEH

WP_046881276.1_(Kv) QSIYQDLDNLRLLADNLARDPRAKITLGCLPSLGLSLVPELVTDFYQQNSNLVMTLTTEH

WP_101997066.1_(Kq) QIIYQDLDNLRLLADNLARDPRAKITLGCLPSLGLSLVPELVTDFYQQNSNLVMTLTTDH

WP_004200266.1_(Ks) QSIYQDLDNLRLLADNLARDPRAKITLGCLPSLGLSLVPELVTDFYQQNSNVVMTLTTEH

WP_109886046.1_(Kp) QSIYQDLDNLRLLADNLARDPRAKITLGCLPSLGLSLVPELVTDFYQQNSNLVMTLTTEH

WP_136032322.1_(Ka) QSIYQDLDNLRLLADNLARDPRAKITLGCLPSLGLSLVPELVTDFYQQNSNLVMTLTTEH

: :*::*. **:**:**:::*:.::::****.***.*:* :.: * ::: *: :*: :.*

KYQ97099.1_(AdmX) TETLVKKLDLREIDLALTMQPVQQGDIMATLIAEVPLVYVDKDYRQGAVEIDSIDQQRWI

WP_070926383.1_(Ps) TVDILQLLTQQDLDIGIGFNLAIEGGITVLPIAEIPLVYVDTEKHQGPISLADIDQERWI

XP_023295947.1_(Lc) TVDILQLLTQQDLDIGIGFNLAIEGGITVLPIAEIPLVYVDTEKHQGPISLADIDQERWI

WP_043083564.1_(Pg) TEMLIKKLALLEIDLALTLQPCEQGDILSHPIADVPLVYIDRDYRQGSVNIEEIDQRRWI

**WP_187509963.1_(Ep)** TETLVKKLDLREIDLALTLQPVQQGEIMATLIAEVPLVYVDKDYRQGAVEIDNIDQQRWI

**WP_158151109.1_(Pa)** TETLVKKLDLREIDLALTLQPVQQGEIMATLIAEVPLVYVDKDYRQGAVEIENIDQQRWI

**WP_116219602.1_(Pa)** TETLVKKLDLREIDLALTLQPVQQGEIMATLIAEVPLVYVDKDYRQGAVEIENIDQQRWI

**WP_062867893.1**_(Sp) TETLVKKLDLREIDLALTMQPVQQGDIMATLIAEVPLVYVDKDYRQGAVEIDSIDQQRWI

**WP_126528367.1**_(Sp) TETLVKKLDLREIDLALTMQPVQQGEIMATLIAEVPLVYVDKDYRQGDVEIDNIDQQRWI

**WP_126483812.1**_(Sp) TETLVKKLDLREIDLALTMQPVQQGEIMATLIAEVPLVYVDKDYRQGDVEIDNIDQQRWI

**WP_062790196.1**_(Sp) TETLVKKLDLREIDLALTMQPIQQGEIIATLIAEVPLVYVDKDYRQGAVEIDNIDQRRWI

**WP_212558166.1_(Sp)** TETLVKKLDLREIDLALTMQPIQQGEIIATLIAEVPLVYVDKDYRQGAVEIDNIDQRRWI

**WP_219227858.1_(Sm)** TETLVKKLDLREIDLALTMQPVEQGEIMATLIAEVPLVYVDKDYRQGAVEIDNIDQQRWI

**WP_103773490.1**_(Sm) TETLVKKLDLREIDLALTMQPVEQGEIMATLIAEVPLVYVDKDYRQGAVEIDNIDQQRWI

**WP_086580121.1**_(Sm) TETLVKKLDLREIDLALTMQPVEQGEIMATLIAEVPLVYVDKDYRQGAVEIDNIDQQRWI

**WP_073532156.1**_(Sm) TETLVKKLDLREIDLALTMQPVQQGEIMATLIAEVPLVYVDKDYRQGAVEIDSIDQQRWI

**WP_046896471.1**_(Se) TETLVKKLDLREIDLALTMQPVEQGEIMATLIAEVPLVYVDKDYRQGAVEIDNVDQQRWI

WP_138096251.1_(Jc) TETLVKKLDLREIDLALTFQPIQQGEITATAIARAPLVYIDRDYRQGAVTMEEIDQQRWI

WP_196611696.1_(Ec) TETLVKKLDLREIDLALTLQPVQQGEIMTTLIAEVPLVYIDRDYRQGAVEIDKIDQQRWI

WP_000776564.1_(Es) TETLVKKLDLREIDLALTLQPIQQGEIMTTLIAEVPLVYIDRDYRQGAVEIDKIDQQRWI

WP_196081520.1_(Ef) TETLVKKLDLREIDLALTLQPIQQGEIMTTLIAEVPLVYIDRDYRQGAVEIDKIDQQRWI

WP_216711806.1_(Ro) SETLVKKLDLREIDLALTLQPVQQGEITSTAIAEVPLVYIDRDYRQGEVAIEEIDQQRWI

WP_143958904.1_(Ro) TETLVKKLDLREIDLALTLQPVQQGEITSTAIAEVPLVYIDRDYRQGEVAIEEIDQQRWI

WP_032714315.1_(Kar) TETLVKKLDLREIDLALTLQPVQQGEIITTLIAEVPLVYIDRDYRQGAVDIKEIDQQRWI

WP_046881276.1_(Kv) TETLVKKLDLREIDLALTLQPVQ---------------------------------QRWI

WP_101997066.1_(Kq) TETLVRKLDLREIDLALTLQPVQQGEILTTLIAEVPLVYIDRHYRQGAVDISQIDQQRWI

WP_004200266.1_(Ks) TETLVKKLDLREIDLALTLQPVQQGEILTTLIAEVPLVYIDRHYRQGAVEIDQIDQQRWI

WP_109886046.1_(Kp) TESLVKKLDLREIDLALTLQPVQQGEILTTLIAEVPLVYIDRHYRQGAVEIDQIDQQRWI

WP_136032322.1_(Ka) TETLIRKLDLREIDLALTLQPVQQGEILTTLIAEVPLVYIDRHYRQGPVEIDQIDQQRWI

: ::: * ::*:.: :: .***

P190 E213 **C215 D232**

KYQ97099.1_(AdmX) SPGLDSLSTAIAAHRVFPATGLNVETCYMAMEFVKRGVGCCITDIFSARHSLTPEMIHQI

WP_070926383.1_(Ps) HPGSDSLSQLLQRYHEFSVSNISVHTYYMAAEFVRAGLGCSITDIFSAEHTLPKSMIYPL

XP_023295947.1_(Lc) HPGSDSLSQLLQRYHEFSVSNISVHTYHMAAEFVRAGLGCSITDIFSAEHTLPKSMIYPL

WP_043083564.1_(Pg) SPGNHSLSSAIAQHRHFSMTRLNVQTYYMATEFVKRGMGCSITDIFSARNNLPPQMIHPL

WP_187509963.1**_(Ep)** SPGPHSLSTVIATRRDFSTTRLNVETCYMATEFVKRGVGCSITDIFTARHNLTPEMIHPI

WP_158151109.1**_(Pa)** SPGPDTLSTFIATRRDFSTTRLNVETCYMATEFVKRGVGCSITDIFTARHNLTPEMIHPI

WP_116219602.1**_(Pa)** SPGPDTLSTFIATRRDFSTTRLNVETCYMATEFVKRGVGCSITDIFTARHNLTPEMIHPI

WP_062867893.1**_(Sp)** SPGLDSLSTAIAAHRVFPATGLNVETCYMAMEFVKRGVGCCITDIFSARHSLTPEMIHQI

WP_126528367.1**_(Sp)** SPGLHSLSSVIATRRVFSTTRLNVETCYMAMEFVKRGVGCSITDIFSARHSLTPEMIHQI

WP_126483812.1**_(Sp)** SPGLHSLSSVIATRRVFSTTRLNVETCYMAMEFVKRGVGCSITDIFSARHSLTPEMIHQI

WP_062790196.1**_(Sp)** SPGLHSVSTAIATRRFFSTTRLNVETCYMAMEFVKRGVGCSITDIFSARHSLTPEMIHQI

**WP_212558166.1_(Sp)** SPGLHSVSTAIATRRFFSTTRLNVETCYMAMEFVKRGVGCSITDIFSARHSLTPEMIHQI

WP_219227858.1**_(Sm)** SPGLHSLSAAIATRRVFSTPRLNVETCYMAMEFVKRGVGCCITDIFSARHTLTPEMIHQI

WP_103773490.1**_(Sm)** SPGLHSLSAAIATRRVFSIPRLNVETCYMAMEFVKRGVGCCITDIFSARHTLTPEMIHQI

WP_086580121.1**_(Sm)** SPGLHSLSAAIATRRVFSIPRLNVETCYMAMEFVKRGVGCCITDIFSARHTLTPEMIHQI

WP_073532156.1**_(Sm)** SPGLHSLSTAIATRRVFSTPRLNVETCYMAMEFVKRGVGCSITDIFSARHSLPPEMIHQI

WP_046896471.1_(Se) SPGLHSLSTAIATRRVFSSPRLNVETCYMAMEFVKRGVGCSITDIFSARHSLPPEMIHQI

WP_138096251.1_(Jc) YPGAHSLSAAIAARRNFATTRLNVQTYYMATEFVKRGMGCSITDIFSARHNLAPQMIHPI

WP_196611696.1_(Ec) SPGPYSLSVAIAKRRDFLTTRLNVQTYYMATEFVKRGMGCSITDIFSARHNLPAETIHPI

WP_000776564.1_(Es) SPGPHSLSNAIAKRRDFLTTRLNVQTYYMATEFVKRGMGCSITDIFSARHNLPAETIHPI

WP_196081520.1_(Ef) SPGPYSLSDAIAKRRDFLTTRLNVQTYYMATEFVKRGMGCSITDIFSARHNLPAETIHPI

WP_216711806.1_(Ro) SPGPHSLSAAIATRRDFSTTRLNVQTYYMATEFVKRGMGCSITDIFSARHNLAPAMIHPI

WP_143958904.1_(Ro) SPGPHSLSAAIATRRDFSTTRLNVQTYYMATEFVKRGMGCSITDIFSARHNLAPAMIHPI

WP_032714315.1_(Kar) SPGPHSLSAAIASRRDFSTTRLNVQTYYMATEFVKRGIGCSITDIFSARHNLSPEMIHPI

WP_046881276.1_(Kv) SPGPHSLSAAIATRRDFSTTRLNVQTYYMATEFVKRGMGCSITDIFSAQHNLDPEMIHPI

WP_101997066.1_(Kq) SPGPHSLSAAIATRRDFSTTRLNVQTYYMATEFVKRGMGCSITDIFSAQHNLAPEMIHPI

WP_004200266.1_(Ks) SPGPHSLSAAIATRRDFSTTRLNVQTYYMATEFVKRGMGCSITDIFSAQHNLAPEMIHPI

WP_109886046.1_(Kp) SPGPHSLSAAIATRRDFSTTRLNVQTYYMATEFVKRGMGCSITDIFSAQHNLAPEMIHPI

WP_136032322.1_(Ka) SPGPHSLSAAIATRRDFSTTRLNVQTYYMATEFVKRGMGCSITDIFSAQHNLAPEMIHPI

** ::* : : * :.*.* :** ***: *:**.*****:*.:.* *: :

KYQ97099.1_(AdmX) SPPMKIDLYLLRRADASLSPVTQKFVDFLCKRLRNELREINLELYPG

WP_070926383.1_(Ps) KENLQLAVSVFHRADRPLTKAAQNYVNTLSQTLEKRNKGVNQKLYSS

XP_023295947.1_(Lc) KENLQLAVSVFHRADRPLTKAAQNYVNTLSQTLEKRNKGVNQKLYSS

WP_043083564.1_(Pg) TPAVKVTLCLLRRSDISLSPVAQKFVDFLCQTLRQQIAAINSELYPE

WP_187509963.1**_(Ep)** SPPMKINLYLLRRADVSLSPVTQKFVDFLCIQLRNQLRVINLELYPD

WP_158151109.1**_(Pa)** SPPMKIDLYLLRRADVSLSPVTHKFVDYLCIQLRNQLRVINQELYPD

WP_116219602.1**_(Pa)** SPPMKIDLYLLRRADVSLSPVTHKFVDYLCIQLRNQLRVINQELYPD

WP_062867893.1**_(Sp)** SPPMKIDLYLLRRADASLSPVTQKFVDFLCKRLRNELREINLELYPG

WP_126528367.1**_(Sp)** SPPMKINLYLLRRADISLSPVTQKFVDFLCKQLRSKLREINLELYPE

WP_126483812.1**_(Sp)** SPPMKINLYLLRRADISLSPVTQKFVDFLCKQLRSKLREVNLELYPE

WP_062790196.1**_(Sp)** SPPMKIDLYLLRRADVSLSPVTQKFVDFLCKQLRSQLKEINLELYPE

**WP_212558166.1_(Sp)** SPPMKIDLYLLRRADVSLSPVTQKFVDFLCKQLRSQLKEINLELYPE

WP_219227858.1**_(Sm)** SPPMKIDLYLLRRADATLSPVTQKFVDFLCKQLRNQLREINLELYPE

WP_103773490.1**_(Sm)** SPPMKIDLYLLRRADATLSPVTQKFVDFLCKQLRNQLREINLELYPE

WP_086580121.1**_(Sm)** SPPMKVDLYLLRRADATLSPVTQKFVDFLCKQLRNQLREINLELYPE

WP_073532156.1**_(Sm)** SPPMKIDLYLLRRSDATLSPVTQKFVDFLCKQLRSQLREINLELYPE

WP_046896471.1_(Se) SPPMKIDLYLLRRADATLSPVTQKFVDFLCKRLRSKLREVNLELYPG

WP_138096251.1_(Jc) TPPLMINLCLLRRADVSLSPITQKFVDFLCQRLRDQLQEINLELYPE

WP_196611696.1_(Ec) EPPMKIDLCLLRRADVSLSPITQKFVDFLCQQLRLQLRAINLELYPE

WP_000776564.1_(Es) EPPMKIDLCLLRRADVSLSPITQKFVDFLCQQLRQQLRAINLELYPE

WP_196081520.1_(Ef) EPPMKIDLCLLRRADVSLSPITQKFVDFLCQQLRLQLRAINLELYPE

WP_216711806.1_(Ro) APPMMITLCLLRRADVSLSPISQKFVDFLCTRLRQQLQEINLELYPE

WP_143958904.1_(Ro) APPMMITLCLLRRADVSLSPISQKFVVFLCTRLRQQLQEINLELYPE

WP_032714315.1_(Kar) TPPMKINLCLLRRADVSLSPITQKFVDFLCKQLRQQLKEINLQLYPD

WP_046881276.1_(Kv) TPPIAINLCLLRRADVSLSPMAQKFVDFLCQRLRQQLKEINLRLYPD

WP_101997066.1_(Kq) TPPMAINLCLLRRADVSLSPMAQKFVDFLCQRLRQQLKEINLRLYPD

WP_004200266.1_(Ks) TPPMAINLCLLRRADVSLSPMAQKFVDFLCQRLRQQLKEINLRLYPD

WP_109886046.1_(Kp) TPPMAINLCLLRRADVSLSPMAQKFVDFLCQRLRQQLKEINLRLYPD

WP_136032322.1_(Ka) TPPIAINLCLLRRADVSLSPMAQKFVDFLCQRLRQQLKEINLRLYPD

: : : :::*:* *: ::::* *. *. . :* .**

# Figure S3: Multiple sequence alignment of AdmX-LBD with ligand binding domains (LBDs) of selected LysR transcriptional regulators. For the selection of LBDs, the AdmX-LBD sequence was used as a query for a BLAST search against the entire RefSeq protein database with the expect threshold set to 0.05. The alignment shows a selection of the 1569 resulting sequences. Residues identified in the crystal structures that establish hydrogen bonds with the indole moiety (blue) and the auxin side chains (red) are highlighted. Amino acids that establish hydrophobic interactions are shown in yellow. Residues numbers refer to full-length AdmX of *S. plymuthica* (Protein GenBank accession KYQ97099.1). The mode by which the highlighted residues interact with IAA and IPA ligands is shown in Fig. 3. Ps, *Providencia stuartii*; Lc, *Lucilia cuprina*; Pg, *Pluralibacter gergoviae*; Ep, *Erwinia persicina*; Pa, *Pantoea ananatis*; Sp, *Serratia plymuthica*; Sm, *Serratia marcescens*; Se, *Serratia* sp.; Jc, *Jejubacter calystegiae*; Ec, *Escherichia coli*; Es, *Escherichia* sp.; Ef, *Escherichia fergusonii*; Ro, *Raoultella ornithinolytica*; Kar, *Klebsiella aerogenes*; Kv, *Klebsiella variicola*; Kq, *Klebsiella quasipneumoniae*; Ks, *Klebsiella* sp.; Kp, *Klebsiella pneumoniae*; Ka, *Klebsiella africana*.
