# Supplementary material for: Emergence of an Auxin Sensing Domain in Plant-Associated Bacteria
Source: mBio. 2023 Jan 5;14(1):e03363-22. doi: 10.1128/mbio.03363-22 (PMC9973260; doi:10.1128/mbio.03363-22)
Supplement: TABLE S5 [file mbio.03363-22-s0010.docx]

**Table S5. Strains and plasmids used in this study.**

| **Strains and plasmids** | **Genotype or relevant characteristics^a^** | **Reference** |
| --- | --- | --- |
| **Strains** | | |
| *Escherichia coli* BL21(DE3) | F^–^ *ompT* *gal* *dcm* *lon* *hsdS_B_*(*r_B_*^–^*m_B_*^–^) λ(DE3 [*lacI* *lacUV5*-*T7p07* *ind1* *sam7* *nin5*]) [*malB*^+^]_K-12_(λ^S^) | (1) |
| *E. coli* BL21-AI | F- *ompT hsdS*_B_ (r_B_^-^m_B_^-^) *gal dcm araB*::*T7RNAP-tetA* | Invitrogen |
| **Plasmids** | | |
| pET28b(+) | Km^R^; Protein expression plasmid. | Novagen |
| pET29b(+) | Km^R^; Protein expression plasmid. | Novagen |
| pMAMV232 | Km^R^; pET29b(+) derivative containing a DNA fragment encoding full length AdmX. C*-*terminal His6*-*tag. | (2) |
| pMAMV235 | Km^R^; pET28b(+) derivative containing a DNA fragment encoding AdmX-LBD (residues 69-295). N*-*terminal His6*-*tag. | (2) |
| pET28-AdmX-LBD_C100S | Km^R^; pET28b(+) derivative containing DNA fragment encoding AdmX-LBD (C100S). N*-*terminal His6*-*tag. | This study^b^ |
| pET28-AdmX-LBD_ E213Q | Km^R^; pET28b(+) derivative containing DNA fragment encoding AdmX-LBD (E213Q). N*-*terminal His6*-*tag. | This study^b^ |
| pET28-AdmX-LBD_C215Y | Km^R^; pET28b(+) derivative containing DNA fragment encoding AdmX-LBD (C215Y). N*-*terminal His6*-*tag. | This study^b^ |
| pET28-WP_109886046.1 | Km^R^; pET28b(+) derivative containing a DNA fragment encoding the LBD of WP_109886046.1 (or AdmX_Kleb) (residues 69-295). N*-*terminal His6*-*tag. | This study^b^ |
| pET28-WP_158151109.1 | Km^R^; pET28b(+) derivative containing a DNA fragment encoding the LBD of WP_158151109.1 (or AdmX_Pan) (residues 69-295). N*-*terminal His6*-*tag. | This study^b^ |
| pET28-WP_187509963.1 | Km^R^; pET28b(+) derivative containing a DNA fragment encoding the LBD of WP_187509963.1 (or AdmX_Erw) (residues 69-295). N*-*terminal His6*-*tag. | This study^b^ |

^a^Km, kanamycin.

^b^Gene synthesis and plasmid construction were done by GenScript Inc.

**REFERENCES**

1. Jeong H, Barbe V, Lee CH, Vallenet D, Yu DS, Choi SH, Couloux A, Lee SW, Yoon SH, Cattolico L, Hur CG, Park HS, Segurens B, Kim SC, Oh TK, Lenski RE, Studier FW, Daegelen P, Kim JF. 2009. Genome sequences of *Escherichia coli* B strains REL606 and BL21(DE3). J Mol Biol 394:644–652.

2. Matilla MA, Daddaoua A, Chini A, Morel B, Krell T. 2018. An auxin controls bacterial antibiotics production. Nucleic Acids Res 46:11229–11238.
